# Supplementary material for: Complex trait methylation scores in the prediction of major depressive disorder
Source: eBioMedicine. 2022 Apr 29;79:104000. doi: 10.1016/j.ebiom.2022.104000 (PMC9062752; doi:10.1016/j.ebiom.2022.104000)
Supplement: Supplementary file 1 [file mmc1.docx]

**Caption for Supplementary Materials**

| **Supplementary File name** | **Supplementary Caption** |
| --- | --- |
| Complex trait MS and MDD Supplementary Materials 24 Feb | Supplementary Materials |
| Complex trait MS and MDD Supplementary Excel File 1 24 Feb | Supplementary Excel File |
| Trait-MS-for-MDD RMarkdown 24 Feb | Supplementary Materials – R code |
