# Supplementary material for: Complex trait methylation scores in the prediction of major depressive disorder
Source: eBioMedicine. 2022 Apr 29;79:104000. doi: 10.1016/j.ebiom.2022.104000 (PMC9062752; doi:10.1016/j.ebiom.2022.104000)
Supplement: Supplementary file 3 [file mmc3.docx]

**Supplementary Materials**

| **Author** | **Trait** | **DNAm array** | **Sample size** | **Participant age, sex** | **Ancestry group** | **Covariates used** |
| --- | --- | --- | --- | --- | --- | --- |
| Braun et al. (2017) | HDL chol | 450K | 725 | 59.9, M=46% | European (Netherlands) | sex, age, smoking, white blood cell proportions, array number, array position |
| Braun et al. (2017) | Total chol | 450K | 725 | 59.9, M=46% | European (Netherlands) | sex, age, smoking, white blood cell proportions, array number, array position |
| Van Dongen et al. (2018) | Education | 450K | 4,152 | Average age and sex across cohorts: 38.2-68.6 years, M=32-47.5% | European (Netherlands) | sex, age at blood sampling, array row, bisulphite plate, white blood cell %, BMI, smoking |
| Liu et al. (2016) | Alcohol consumption | 450K | 13,317 | Average age and sex across cohorts: 42-76 years, M=46% | European (N=9643), African (N=2023), Multi-ethnic, monocyte (N=1251) | age, sex, BMI, batch, white cell blood counts, smoking status |
| Wahl et al. (2016) | BMI | 450K | 5,387 | Average age and sex across cohorts: 51-61 years, M=48.9-67.7% | EPICOR, KORA: European; LOLIPOP: Indian Asian | age, sex, smoking status, physical activity index, alcohol consumption, first 20 PCs, estimated white blood cell proportions |
| Joehanes et al. (2016) | Smoking status | 450K | 15,907 | Current: 57.7, M=46.3%; Former: 64.8, M=55.6%; Never: 61.2, M=31.7% | N=12161 European; N=3746 African American | age, sex, blood count, technical covariates |

**Supplementary Table 1.** MWAS demographic information; Article DOI: HDL and total cholesterol: <https://doi.org/10.1186/s13148-016-0304-4> (1); educational attainment: <https://doi.org/10.1038/s41539-018-0020-2> (2); alcohol consumption: <https://doi.org/10.1038/mp.2016.192> (3); BMI: <https://doi.org/10.1038/nature20784> (4);smoking status: <https://doi.org/10.1161/circgenetics.116.001506> (5).

**Additional sample information for ALSPAC**

Pregnant women resident in Avon, UK with expected dates of delivery 1st April 1991 to 31st December 1992 were invited to take part in the study. The initial number of pregnancies enrolled is 14,541 (for these at least one questionnaire has been returned or a “Children in Focus” clinic had been attended by 19/07/99). Of these initial pregnancies, there was a total of 14,676 foetuses, resulting in 14,062 live births and 13,988 children who were alive at 1 year of age.

When the oldest children were approximately 7 years of age, an attempt was made to bolster the initial sample with eligible cases who had failed to join the study originally. As a result, when considering variables collected from the age of seven onwards (and potentially abstracted from obstetric notes) there are data available for more than the 14,541 pregnancies mentioned above. The number of new pregnancies not in the initial sample (known as Phase I enrolment) that are currently represented on the built files and reflecting enrolment status at the age of 24 is 913 (456, 262 and 195 recruited during Phases II, III and IV respectively), resulting in an additional 913 children being enrolled. The phases of enrolment are described in more detail in the cohort profile paper and its update (see footnote 4 below). The total sample size for analyses using any data collected after the age of seven is therefore 15,454 pregnancies, resulting in 15,589 foetuses. Of these 14,901 were alive at 1 year of age.

A 10% sample of the ALSPAC cohort, known as the Children in Focus (CiF) group, attended clinics at the University of Bristol at various time intervals between 4 to 61 months of age. The CiF group were chosen at random from the last 6 months of ALSPAC births (1432 families attended at least one clinic). Excluded were those mothers who had moved out of the area or were lost to follow-up, and those partaking in another study of infant development in Avon.

**Phenotype measurement GS:SFHS**

The SCID was administered to participants who answered “yes” to either of the following screening questions: “Have you ever seen anybody for emotional or psychiatric problems?” and “Was there ever a time when you, or someone else, thought you should see someone because of the way you were feeling or acting?”. Answers from the SCID were used to ascertain MDD case status.

Educational attainment was recorded by asking participants: “What is the highest educational qualification you have obtained?”, with the following possible answers: 1 - College or University degree, 2 - Other professional or technical qualification, 3 - NVQ or HND or HNC or equivalent, 4 - Higher Grade, A levels, AS levels or equivalent, 5 - Standard Grade, O levels, GCSEs or equivalent, 6 - CSEs or equivalent, 7 - School leavers certificate, 8 - Other, 9 - No Qualification.

**Phenotype measurement in ALSPAC**

Alcohol consumption was measured by asking participants the frequency of alcohol consumption, with the following possible answers: 1 – Never, 2 – Monthly or less, 3 – 2 to 4 times a month, 4 – 2 to 3 times a week, and 5 – 4 or more times a week.

Educational attainment was recorded by asking participants: “What is the highest educational qualification you have obtained?”, with the following possible answers**:** 0 – None, 1 – CSE, 2 – Vocational, 3 – O-level, 4 – A-level, 5 – Degree. This variable was collected when mothers had a mean age of ~29, at 32 weeks gestation, and participants may have different levels of education at the time of blood draw (mean age= ~47).

**Methylation principal components in GS:SFHS and ALSPAC**

In GS:SFHS, methylation PCs were regressed out during pre-processing and quality checking of the DNA methylation data. The first 10 methylation PCs explained 97.7% of the variance in control probes.

In ALSPAC, methylation PCs were calculated from M-values that had been pre-corrected for age, sex, estimated cell counts, processing batch and relatedness using the R package FactoMineR (6).

Correlations between PCs in GS:SFHS and ALSPAC and continuous covariates included in regression models are shown in Supplementary Tables 2 and 3 below.

|  | **Alcohol units** | **BMI** | **Pack years** |
| --- | --- | --- | --- |
| PC1 | 0.011 | -0.007 | -0.021 |
| PC2 | -0.002 | 0.002 | -0.015 |
| PC3 | 0.022 | -0.005 | -0.009 |
| PC4 | 0.059 | 0.002 | 0.025 |
| PC5 | 0.016 | -0.01 | -0.006 |
| PC6 | 0.333 | 0.053 | 0.05 |
| PC7 | 0.08 | 0.022 | 0.045 |
| PC8 | -0.003 | 0.001 | -0.005 |
| PC9 | 0.001 | 0.018 | 0.005 |
| PC10 | -0.03 | -0.026 | -0.005 |

**Supplementary Table 2.** Correlations between 10 methylation PCs and continuous lifestyle factors in GS:SFHS (N=9,502).

|  | **Bcell** | **CD4T** | **CD8T** | **Gran** | **NK** | **BMI** |
| --- | --- | --- | --- | --- | --- | --- |
| PC1 | 0.384 | 0.043 | -0.099 | -0.203 | 0.218 | -0.016 |
| PC2 | 0.001 | 0.098 | -0.023 | -0.046 | 0.057 | -0.056 |
| PC3 | 0.237 | 0.414 | 0.167 | -0.502 | 0.404 | 0.014 |
| PC4 | -0.127 | -0.212 | -0.206 | 0.3 | -0.212 | -0.001 |
| PC5 | 0.035 | 0.088 | 0.016 | -0.003 | -0.07 | -0.035 |
| PC6 | -0.053 | -0.187 | 0.044 | 0.137 | -0.061 | -0.068 |
| PC7 | 0.182 | 0.173 | -0.06 | -0.144 | 0.091 | 0.011 |
| PC8 | -0.123 | -0.255 | 0.068 | 0.215 | -0.091 | -0.004 |
| PC9 | 0.011 | 0.199 | 0.038 | -0.123 | -0.023 | -0.029 |
| PC10 | -0.088 | -0.074 | 0.045 | 0.041 | -0.006 | -0.027 |
| PC11 | -0.074 | -0.098 | 0.072 | 0.098 | -0.059 | 0.001 |
| PC12 | -0.0002 | -0.009 | 0.042 | -0.017 | 0.037 | 0.053 |
| PC13 | -0.09 | -0.148 | 0.091 | 0.083 | -0.049 | -0.063 |
| PC14 | -0.001 | 0.008 | 0.071 | -0.05 | 0.005 | -0.05 |
| PC15 | -0.059 | -0.076 | 0.004 | 0.098 | -0.07 | 0.027 |
| PC16 | 0.017 | 0.044 | 0.02 | -0.012 | -0.037 | -0.074 |
| PC17 | -0.021 | 0.019 | 0.062 | -0.008 | -0.031 | 0.089 |
| PC18 | -0.01 | -0.033 | -0.001 | 0.037 | 0.01 | 0.061 |
| PC19 | 0.021 | -0.014 | -0.008 | 0.003 | 0.033 | 0.072 |
| PC20 | 0.015 | -0.008 | -0.029 | 0.043 | 0.005 | 0.025 |

**Supplementary Table 3.** Correlations between 10 methylation PCs and continuous lifestyle factors in ALSPAC (N=565).

| **Trait** | **Beta** | **P-value** |
| --- | --- | --- |
| Glucose | -0.0023 | 0.9375 |
| **HDL cholesterol** | **-0.1159** | **0.0001** |
| **Total cholesterol** | **0.0689** | **0.0164** |
| **Sodium** | **-0.1062** | **0.0004** |
| **Potassium** | **0.0668** | **0.0111** |
| **Urea** | **-0.1485** | **2.28E-05** |
| Creatinine | 0.0171 | 0.6135 |
| Creatinine mgdl | 0.0120 | 0.7225 |
| Height | -0.0211 | 0.6017 |
| **Weight** | **0.1499** | **1.91E-07** |
| **Waist** | **0.2010** | **1.89E-12** |
| **Hips** | **0.1919** | **2.51E-13** |
| **Waist:hip ratio** | **0.0875** | **0.0046** |
| **Body fat** | **0.2060** | **1.47E-08** |
| **SIMD quintile** | **-0.1037** | **1.80E-07** |
| **SIMD rank** | **-0.1490** | **9.98E-08** |
| Educational attainment | -0.0035 | 7.66E-01 |
| **Drink status** | **0.1562** | **3.65E-05** |
| **Alcohol consumption (units)** | **0.1026** | **0.0007** |
| **BMI** | **0.1488** | **1.00E-08** |
| **Smoking status** | **-0.2157** | **7.32E-20** |
| **Pack years** | **0.1554** | **7.87E-09** |
| **Neuroticism Total** | **0.2739** | **1.35E-181** |
| **Extraversion Total** | **-0.0777** | **1.21E-21** |

**Supplementary Table 4.** Associations between environmental, lifestyle, and biochemical factors and MDD in GS (N=9,502). Rows in bold are variables significantly associated with MDD. Previous well-powered MWAS exist for: HDL and total cholesterol; smoking status; alcohol consumption (units); BMI; educational attainment.

**Literature search to identify previous MWAS**

Google Scholar, Scopus, and Web of Science were searched in January 2021 to identify studies that investigated DNAm signatures of factors that were significantly associated with MDD in our analyses. There was no year limit imposed in the search strategy. The search comprised OR clusters containing key terms, which were combined with AND. Key terms included, but were not limited to:

**DNA methylation, epigen*, EWAS, epigenome-wide association study, lifestyle, environmental, as well as all terms included in the “Trait” column in Supplementary Table 2.**

| **Trait** | **N CpGs identified in MWAS** | **N CpGs present in GS:SFHS** | **N CpGs present in ALSPAC** |
| --- | --- | --- | --- |
| HDL cholesterol | 56 | 49 | 56 |
| Total cholesterol | 4 | 4 | 4 |
| BMI | 278 | 260 | 276 |
| Educational attainment |  |  |  |
| MW | 11 | 8 | 11 |
| 0.01 | 6,505 | 5,201 | 6,489 |
| 0.05 | 26,827 | 21,230 | 26,728 |
| 0.1 | 49,853 | 39,152 | 49,662 |
| 0.5 | 214,848 | 166,990 | 213,827 |
| Smoking status |  |  |  |
| MW | 18,760 | 15,814 | 18,029 |
| 0.01 | 45,152 | 37,457 | 43,284 |
| 0.05 | 80,130 | 64,072 | 76,249 |
| 0.1 | 110,546 | 86,520 | 104,776 |
| 0.5 | 282,775 | 210,386 | 265,566 |
| Alcohol consumption |  |  |  |
| MW | 363 | 328 | 363 |
| 0.01 | 18,199 | 15,031 | 17,450 |
| 0.05 | 45,280 | 35,892 | 42,861 |
| 0.1 | 72,082 | 55,672 | 67,797 |
| 0.5 | 258,988 | 187,045 | 240,123 |

**Supplementary Table 5.** Overlap between CpGs identified in previous MWAS and CpGs present in GS and ALSPAC, used to construct MS.; MW=methylome-wide.

| **Demographic characteristic** | **MDD diagnosis (N=408)** | **No MDD Diagnosis (N=4,024)** |
| --- | --- | --- |
| Age (mean, SD) | 47.67 (11.94) | 51.63 (13.15) |
| Sex (%) | F=285 (70%) | F=2,496 (62%%) |
| BMI (mean, SD) | 27.05 (5.49) | 26.56 (4.69) |
| Alcohol units (mean, SD) | 11.15 (14.34) | 10.68 (11.25) |
| Smoking status |  |  |
| Current smoker | 125 | 553 |
| Former smokers (quit < 1 year ago) | 9 | 107 |
| Former smokers (quit > 1 year ago) | 106 | 1,197 |
| Never smoked tobacco | 163 | 2,117 |
| Pack years (mean, SD) | 9.92 (15.98) | 6.96 (13.37) |
| Educational attainment |  |  |
| Any qualification | 327 | 3,375 |
| No qualification | 43 | 331 |
| Other | 13 | 94 |
| HDL cholesterol (mean, SD) | 1.48 (0.41) | 1.47 (0.41) |
| Total cholesterol (mean, SD) | 5.16 (1.1) | 5.21 (1.05) |

**Supplementary Table 6**. Demographic characteristics for individuals with an MDD diagnosis and controls in GS:SFHS subset (N=4,432).


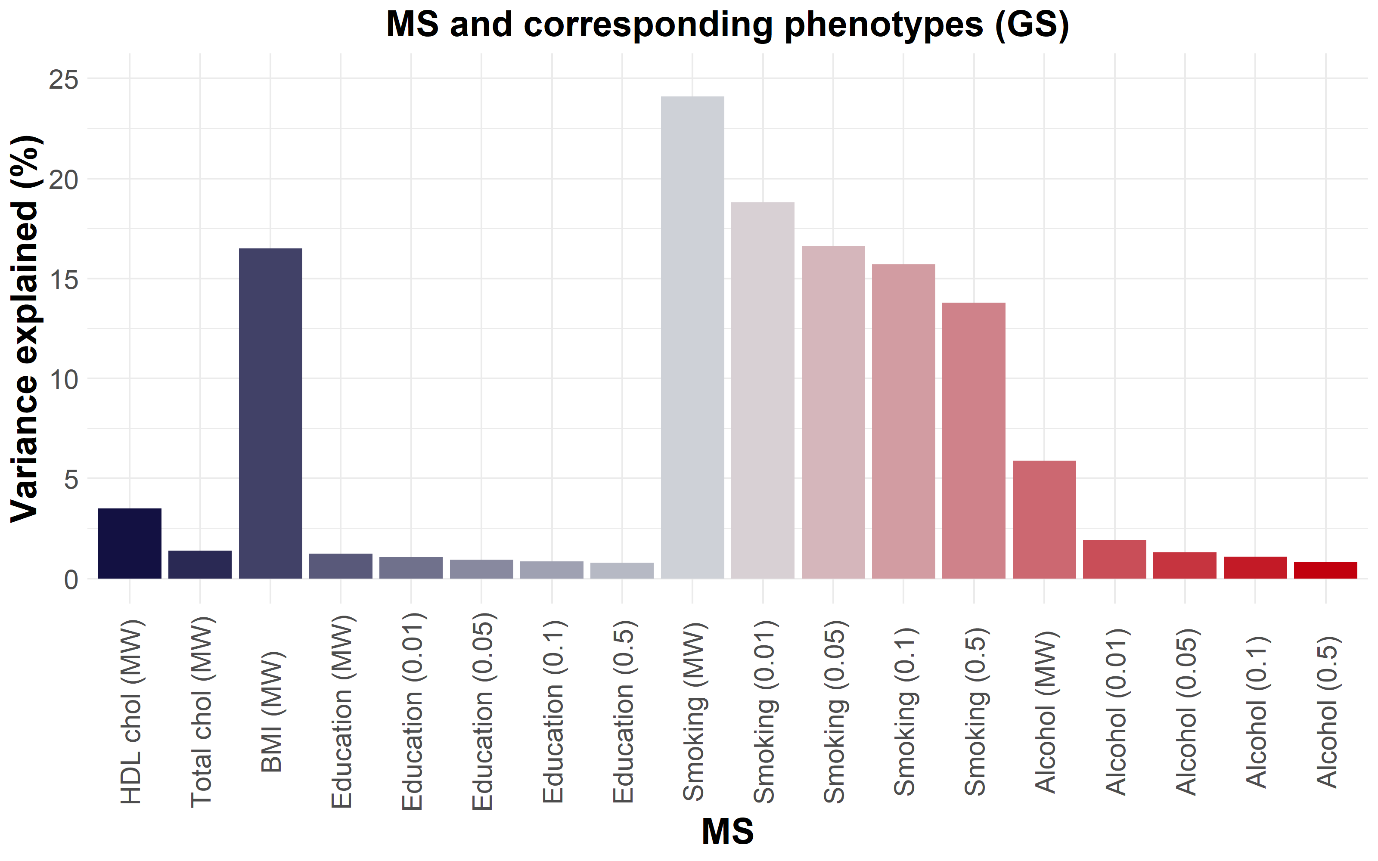


**Supplementary Figure 1.** GS: variance explained (indicated by R^2^ (%) on the y-axis) by each MS in their corresponding phenotype. For each model, the phenotype was fitted as the outcome variable with sex and age as covariates; each MS was fitted as a predictor variable. Where available, R^2^ is calculated for MS at different thresholds (educational attainment, smoking status, alcohol consumption). MW=methylome-wide.

**
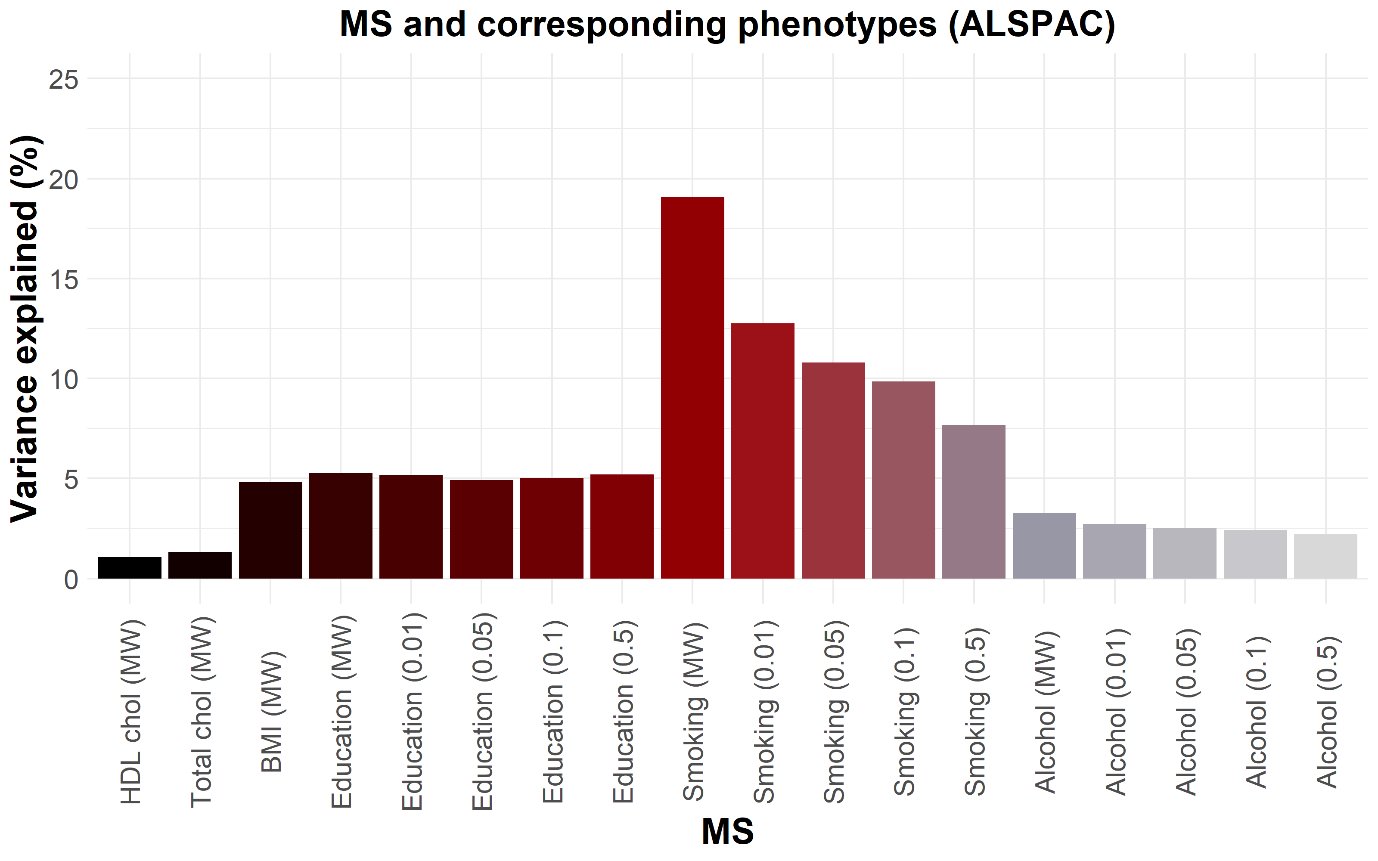
**

**Supplementary Figure 2.** ALSPAC: variance explained (indicated by R^2^ (%) on the y-axis) by each MS in their corresponding phenotype. For each model, the phenotype was fitted as the outcome variable with age, 20 methylation PCs, and 5 cell types as covariates; each MS was fitted as a predictor variable. Where available, R^2^ is calculated for MS at different thresholds (educational attainment, smoking status, alcohol consumption). MW=methylome-wide.

|  | **Model 1** | **Model 2** | **Model 3** |
| --- | --- | --- | --- |
| **MS** | **R^2^ (%)** | | |
| **HDL cholesterol (MW)** | **0.2%** | **0.1%** | 0.05% |
| **Total cholesterol (MW)** | **0.09%** | **0.1%** | 0.03% |
| **BMI (MW)** | **0.3%** | **0.1%** | 0.03% |
| **Educational attainment** |  |  |  |
| MW | **0.3%** | **0.28%** | **0.06%** |
| 0.01 | **0.3%** | **0.3%** | 0.02% |
| 0.05 | **0.2%** | **0.2%** | 0.02% |
| 0.1 | **0.2%** | **0.2%** | 0.02% |
| 0.5 | **0.2%** | **0.2%** | 0.03% |
| **Smoking status** |  |  |  |
| MW | **0.36%** | 0.03% | 0.01% |
| 0.01 | **0.35%** | **0.06%** | 0.03% |
| 0.05 | **0.35%** | **0.07%** | 0.03% |
| 0.1 | **0.34%** | **0.07%** | 0.03% |
| 0.5 | **0.31%** | **0.07%** | 0.03% |
| **Alcohol units** |  |  |  |
| MW | **0.08%** | **0.05%** | 0.004% |
| 0.01 | **0.08%** | **0.06%** | **0.07%** |
| 0.05 | **0.08%** | **0.1%** | **0.09%** |
| 0.1 | **0.08%** | **0.1%** | **0.09%** |
| 0.5 | **0.2%** | **0.1%** | **0.09%** |

**Supplementary Table 7.** R^2^ for model 1 (covariates: age, sex), model 2 (covariates: model 1 + each MS’s corresponding phenotype), and model 3 (model 2 + 4 lifestyle factors, BMI, smoking, pack years, and alcohol consumption) in GS (N=9,502). Each MS was associated with MDD. Where available (educational attainment, smoking status, alcohol units), associations are presented for MS calculated at multiple thresholds (p=methylome-wide, <0.01, <0.05, <0.1, <0.5). MW=methylome-wide. Statistically significant results are represented in bold.

|  | **Model 1** | **Model 2** | **Model 3** |
| --- | --- | --- | --- |
| **MS** | **R^2^ (%)** | | |
| **HDL cholesterol (MW)** | 0.02% | 0 | 1.33% |
| **Total cholesterol (MW)** | 0 | 0 | 0 |
| **BMI (MW)** | 0 | 0 | 0 |
| **Educational attainment** |  |  |  |
| MW | 0.46% | 0.14% | 0 |
| 0.01 | 0.44% | 0.18% | 0.35% |
| 0.05 | 0.77% | 0.51% | 0.75% |
| 0.1 | 0.87% | 0.57% | 0.77% |
| 0.5 | **1.42%** | 0.97% | 1.51% |
| **Smoking status** |  |  |  |
| MW | 0.24% | 0.06% | 0.586% |
| 0.01 | 0.13% | 0.01% | 0.378% |
| 0.05 | 0.03% | 0 | 0.164% |
| 0.1 | 0 | 0 | 0.073% |
| 0.5 | 0 | 0 | 0 |
| **Alcohol units** |  |  |  |
| MW | 0.40% | 0.32% | **1.376%** |
| 0.01 | 0 | 0 | 0.422% |
| 0.05 | 0 | 0 | 0.346% |
| 0.1 | 0 | 0 | 0.327% |
| 0.5 | 0 | 0 | 0.272% |

**Supplementary Table 8.** R^2^ for model 1 (covariates: age, 20 methylation PCs, and 5 cell types), model 2 (covariates: model 1 + each MS’s corresponding phenotype), and model 3 (model 2 + 3 lifestyle factors, BMI, smoking, and alcohol consumption) in ALSPAC (N=565). Each MS was associated with MDD. Where available (educational attainment, smoking status, alcohol units), associations are presented for MS calculated at multiple thresholds (p=methylome-wide, <0.01, <0.05, <0.1, <0.5). MW=methylome-wide. Statistically significant results are represented in bold.

|  | **Model 2** | | | **Model 3** | | |
| --- | --- | --- | --- | --- | --- | --- |
| **MS** | **Beta** | **P-value** | **R^2^ (%)** | **Beta** | **P-value** | **R^2^ (%)** |
| **HDL cholesterol (MW)** | **-0.145** | **0.00002** | **0.326%** | **-0.113** | **0.002** | **0.192%** |
| **Total cholesterol (MW)** | **-0.093** | **0.005** | **0.138%** | -0.045 | 0.219 | 0.031% |
| **BMI (MW)** | **0.099** | **0.006** | **0.134%** | 0.064 | 0.109 | 0.052% |
| **Educational attainment** |  |  |  |  |  |  |
| MW | **-0.135** | **0.0001** | **0.28%** | **0.154** | **0.007** | **0.146%** |
| 0.01 | **-0.139** | **0.0001** | **0.292%** | -0.023 | 0.582 | 0.007% |
| 0.05 | **-0.098** | **0.006** | **0.152%** | -0.007 | 0.867 | 0.0006% |
| 0.1 | **-0.091** | **0.01** | **0.131%** | -0.009 | 0.827 | 0.0009% |
| 0.5 | **-0.083** | **0.018** | **0.112%** | -0.014 | 0.715 | 0.003% |
| **Smoking status** |  |  |  |  |  |  |
| MW | 0.044 | 0.263 | 0.027% | 0.003 | 0.951 | 0.0004% |
| 0.01 | 0.07 | 0.062 | 0.07% | 0.034 | 0.408 | 0.019% |
| 0.05 | **0.078** | **0.035** | **0.087%** | 0.042 | 0.291 | 0.029% |
| 0.1 | **0.08** | **0.029** | **0.094%** | 0.045 | 0.253 | 0.033% |
| 0.5 | **0.081** | **0.025** | **0.098%** | 0.047 | 0.226 | 0.037% |
| **Alcohol units** |  |  |  |  |  |  |
| MW | 0.005 | 0.903 | 0.0006% | -0.059 | 0.135 | 0.04% |
| 0.01 | **-0.128** | **0.0006** | **0.252%** | **-0.139** | **0.0003** | **0.292%** |
| 0.05 | **-0.148** | **0.00007** | **0.342%** | **-0.148** | **0.0001** | **0.337%** |
| 0.1 | **-0.154** | **0.00003** | **0.373%** | **-0.15** | **0.00008** | **0.348%** |
| 0.5 | **-0.161** | **0.00001** | **0.412%** | **-0.151** | **0.00007** | **0.356%** |

**Supplementary Table 9.** Associations between MDD and environmental MS in a women-only subsample in GS (N=5,615) for model 2 (covariates: age, each MS’s corresponding phenotype) and model 3 (model 2 + 4 lifestyle factors, BMI, smoking, pack years, and alcohol consumption). Where available (educational attainment, smoking status, alcohol units), associations are presented for MS calculated at multiple thresholds (p=methylome-wide, <0.01, <0.05, <0.1, <0.5). MW=methylome-wide. Statistically significant results are represented in bold.

|  | **Smoking status not included** | | **Smoking status included** | |
| --- | --- | --- | --- | --- |
|  | **GS** | | | |
| **MS** | **Beta** | **P-value** | **Beta** | **P-value** |
| **HDL cholesterol (MW)** | **-0.113** | **3.81x10^-5^** | **-0.104** | **0.0002** |
| **Total cholesterol (MW)** | **-0.077** | **0.005** | **-0.061** | **0.031** |
| **BMI (MW)** | **0.138** | **3.83x10^-7^** | **0.106** | **0.0001** |
| **Educational attainment** |  |  |  |  |
| MW | **-0.142** | **9.77x10^-8^** | 0.065 | 0.096 |
| 0.01 | **-0.148** | **7.25 x10^-8^** | -0.059 | 0.051 |
| 0.05 | **-0.125** | **6.30 x10^-6^** | -0.049 | 0.097 |
| 0.1 | **-0.120** | **1.65 x10^-5^** | -0.049 | 0.093 |
| 0.5 | **-0.109** | **7.37 x10^-5^** | -0.048 | 0.100 |
| **Alcohol units** |  |  |  |  |
| MW | **0.061** | **0.03** | 0.006 | 0.826 |
| 0.01 | **-0.061** | **0.03** | **-0.089** | **0.002** |
| 0.05 | **-0.083** | **0.004** | **-0.100** | **0.0005** |
| 0.1 | **-0.089** | **0.002** | **-0.102** | **0.0004** |
| 0.5 | **-0.097** | **0.0006** | **-0.105** | **0.0003** |
|  | **ALSPAC** | | | |
| **MS** | **Beta** | **P-value** | **Beta** | **P-value** |
| **HDL cholesterol (MW)** | -0.149 | 0.557 | -0.145 | 0.571 |
| **Total cholesterol (MW)** | 0.029 | 0.855 | 0.038 | 0.813 |
| **BMI (MW)** | -0.04 | 0.823 | -0.034 | 0.850 |
| **Educational attainment** |  |  |  |  |
| MW | -0.230 | 0.064 | -0.209 | 0.246 |
| 0.01 | -0.231 | 0.195 | -0.171 | 0.356 |
| 0.05 | -0.330 | 0.105 | -0.275 | 0.188 |
| 0.1 | -0.359 | 0.092 | -0.306 | 0.160 |
| 0.5 | **-0.483** | **0.03** | -0.433 | 0.057 |
| **Alcohol units** |  |  |  |  |
| MW | 0.283 | 0.156 | 0.244 | 0.228 |
| 0.01 | -0.03 | 0.899 | -0.043 | 0.856 |
| 0.05 | -0.09 | 0.715 | -0.089 | 0.720 |
| 0.1 | -0.096 | 0.698 | -0.090 | 0.719 |
| 0.5 | -0.099 | 0.688 | -0.084 | 0.734 |

**Supplementary Table 10.** Model 1 in GS (N=9,502) and ALSPAC (N=565) when excluding/including smoking status as a covariate, in addition to age and sex (GS only). Comparisons are made for HDL and total cholesterol, BMI, educational attainment, and alcohol units. Where available (educational attainment, alcohol units), associations are presented for MS calculated at multiple thresholds (p=methylome-wide (Bonferroni-corrected CpGs), <0.01, <0.05, <0.1, <0.5). MW=methylome-wide. Statistically significant results are represented in bold.

| **CpG site** | **Beta** |
| --- | --- |
| cg12609142 | -0.01119 |
| cg16145216 | 0.058131 |
| cg18289580 | 0.146244 |
| cg09935388 | -0.03242 |
| cg16054275 | -0.18677 |
| cg01730064 | 0.014476 |
| cg02335251 | -0.00532 |
| cg26253134 | -0.01633 |
| cg07258149 | -0.02817 |
| cg26839652 | 0.133703 |
| cg14639163 | 0.260279 |
| cg12934382 | 0.128504 |
| cg12405098 | 0.129092 |
| cg17795252 | 0.023548 |
| cg15233074 | -0.09448 |
| cg02419901 | -0.102 |
| cg19216561 | -0.07034 |
| cg03970609 | 0.005039 |
| cg08936411 | 0.0456 |
| cg05052463 | 0.492047 |
| cg13999210 | 0.067257 |
| cg22479161 | -0.0053 |
| cg14753356 | -0.06617 |
| cg00124375 | 0.044034 |
| cg17983217 | 0.03929 |
| cg17508500 | 0.043798 |
| cg06340118 | -0.07493 |
| cg20768122 | 0.054116 |
| cg06589246 | 0.033978 |
| cg22400291 | 0.048691 |
| cg17019350 | 0.04029 |
| cg11022926 | 0.015782 |
| cg26424649 | 0.004055 |
| cg14400718 | 0.008611 |
| cg20984053 | -0.18912 |
| cg03295542 | 0.024814 |
| cg10034808 | -0.00317 |
| cg13021857 | -0.15757 |
| cg26054672 | -0.10536 |
| cg01692968 | -0.20543 |
| cg26135388 | -0.04544 |
| cg23190089 | -0.09462 |
| cg06533728 | -0.0001 |
| cg18975055 | -0.03513 |
| cg13529291 | 0.012074 |
| cg03747614 | -0.02455 |
| cg10148841 | -0.02582 |
| cg21745586 | -0.05385 |
| cg21486233 | 0.038797 |
| cg23146197 | -0.16761 |
| cg07258897 | -0.04571 |
| cg15948377 | 0.028911 |
| cg25543578 | 0.042259 |
| cg11101030 | -0.04732 |
| cg17494199 | -0.23309 |
| cg05514401 | -0.10268 |
| cg18908185 | -0.06826 |
| cg14053764 | 0.110107 |
| cg26009013 | -0.20612 |
| cg13662173 | -0.04893 |
| cg07084930 | -0.14777 |
| cg00552822 | 0.088955 |
| cg26889953 | -0.16039 |
| cg15721541 | -0.04605 |
| cg09552652 | -0.03886 |
| cg01536682 | 0.034552 |
| cg05828191 | -0.01444 |
| cg16531275 | -0.06062 |
| cg25579860 | -0.00655 |
| cg21848117 | 0.139987 |
| cg13280079 | -0.08524 |
| cg13674271 | -0.00068 |
| cg23321951 | -0.03603 |
| cg07083818 | -0.00833 |
| cg03346025 | -0.74837 |
| cg25721806 | -0.00998 |
| cg12033214 | -0.06256 |
| cg00187244 | 0.012398 |

**Supplementary Table 11.** List of CpG sites (N=78) comprising the MDD MS derived from LASSO (N_participants_=5,078).

**References**

1. Braun KVE, Dhana K, de Vries PS, Voortman T, van Meurs JBJ, Uitterlinden AG, et al. Epigenome-wide association study (EWAS) on lipids: the Rotterdam Study. Clin Epigenetics. 2017 Feb;9(1):15.

2. van Dongen J, Bonder MJ, Dekkers KF, Nivard MG, van Iterson M, Willemsen G, et al. DNA methylation signatures of educational attainment. npj Sci Learn. 2018 Dec;3(1):7.

3. Liu C, Marioni RE, Hedman ÅK, Pfeiffer L, Tsai P, Reynolds LM, et al. A DNA methylation biomarker of alcohol consumption. Mol Psychiatry. 2018;(February 2016):422–33.

4. Wahl S, Drong A, Lehne B, Loh M, Scott WR, Kunze S, et al. Epigenome-wide association study of body mass index, and the adverse outcomes of adiposity. Nature. 2017 Jan;541(7635):81–6.

5. Joehanes R, Just AC, Marioni RE, Pilling LC, Reynolds LM, Mandaviya PR, et al. Epigenetic Signatures of Cigarette Smoking. Circ Cardiovasc Genet. 2016 Oct;9(5):436–47.

6. Lê S, Josse J, Husson F. FactoMineR: An R Package for Multivariate Analysis. J Stat Softw [Internet]. 2008 Mar 18 [cited 2022 Feb 25];25(1):1–18. Available from: https://www.jstatsoft.org/index.php/jss/article/view/v025i01
