# Supplementary material for: Complex trait methylation scores in the prediction of major depressive disorder
Source: eBioMedicine. 2022 Apr 29;79:104000. doi: 10.1016/j.ebiom.2022.104000 (PMC9062752; doi:10.1016/j.ebiom.2022.104000)

Trait MS for MDD Rscript

Miruna Barbu

25/03/2021

## Trait MS for MDD Rscript

The following R Markdown project outlines the code used to generate data and analysis for the project entitled, “Trait methylation scores in the prediction of major depressive disorder”.

### Install all libraries needed for project

library(readxl)
library(MuMIn)
library(ggplot2)
library(ggpubr)
library("pROC")

## Type 'citation("pROC")' for a citation.

##
## Attaching package: 'pROC'

## The following objects are masked from 'package:stats':
##
## cov, smooth, var

library(stringr)
library(data.table)
library(gameofthrones)
library(tidyr)
library(dplyr)

##
## Attaching package: 'dplyr'

## The following objects are masked from 'package:data.table':
##
## between, first, last

## The following objects are masked from 'package:stats':
##
## filter, lag

## The following objects are masked from 'package:base':
##
## intersect, setdiff, setequal, union

library(readxl)
library(coloc)

##
## Attaching package: 'coloc'

## The following object is masked from 'package:pROC':
##
## ci

library(locuscomparer)
library(devtools)

## Loading required package: usethis

#install.packages("remotes")
#remotes::install_github("MRCIEU/gwasglue")
#devtools::install_github("MRCIEU/gwasglue")

### Data preparation

The following section consists of data preparation for GS:SFHS main analysis. We sorted through all individuals with DNAm data to obtain a final dataset to be used in (1) MS calculation and (2) association between MS and a number of variables

# wave dep_status sex age units bmi
# 1:5070 0:7876 F:5615 Min. :18.00 Min. : 0.0 Min. :14.78
# 3:4432 1:1626 M:3887 1st Qu.:41.00 1st Qu.: 2.0 1st Qu.:23.29
# Median :52.00 Median : 8.0 Median :26.11
# Mean :49.83 Mean : 10.6 Mean :26.89
# 3rd Qu.:59.00 3rd Qu.: 15.0 3rd Qu.:29.51
# Max. :95.00 Max. :326.0 Max. :67.62
# NA's :847 NA's :53
# ever_smoke HDL_cholesterol Total_cholesterol pack_years
# 1 :1610 Min. :0.400 Min. : 0.900 Min. : 0.000
# 2 : 274 1st Qu.:1.200 1st Qu.: 4.400 1st Qu.: 0.000
# 3 :2609 Median :1.400 Median : 5.100 Median : 0.000
# 4 :4801 Mean :1.478 Mean : 5.166 Mean : 7.905
# NA's: 208 3rd Qu.:1.700 3rd Qu.: 5.900 3rd Qu.: 10.000
# Max. :4.500 Max. :12.000 Max. :133.000
# NA's :83 NA's :67 NA's :207

### Regression models - MDD and phenotypes in GS:SFHS

MDD-relevant factor selection - which phenotypes are associated with MDD in GS:SFHS? Covariates included in all models: age, sex

# Scale continuous variables:
lifestyle_mdd[,c(10,15:30)] <- scale(lifestyle_mdd[,c(10,15:30)])
n <-31 # Change according to number of lifestyle variables to include
glm_mdd <- lapply(9:n, function(x) glm(dep_status ~ age+sex+lifestyle_mdd[,x],data=lifestyle_mdd,family="binomial"))
summary_glm_mdd <- lapply(glm_mdd, summary)

stats_glm_mdd = lapply(summary_glm_mdd, function(x) x$coefficients[c(4),])
names(stats_glm_mdd) = colnames(lifestyle_mdd)[9:31] # Change according to scores
stats_dataframe = data.frame(stats_glm_mdd)

stats_dataframe = t(stats_dataframe)
stats_dataframe = data.frame(stats_dataframe)
print(stats_dataframe)

## Estimate Std..Error z.value Pr...z..
## drink_status 1.561810e-01 3.782696e-02 4.12882709 3.646185e-05
## units 1.026165e-01 3.022794e-02 3.39475576 6.868985e-04
## NeuroticismTotal 2.739482e-01 9.533271e-03 28.73601829 1.354427e-181
## ExtraversionTotal -7.773147e-02 8.133385e-03 -9.55708784 1.211168e-21
## SIMD_quintile -1.037156e-01 1.987295e-02 -5.21893420 1.799557e-07
## SIMD_rank -7.967538e-05 1.495681e-05 -5.32703011 9.983162e-08
## height -2.111507e-02 4.045163e-02 -0.52198313 6.016821e-01
## weight 1.498785e-01 2.877656e-02 5.20835409 1.905231e-07
## bmi 1.487509e-01 2.595817e-02 5.73040563 1.001908e-08
## waist 2.010454e-01 2.854814e-02 7.04232940 1.890522e-12
## hips 1.919284e-01 2.622590e-02 7.31827892 2.511714e-13
## whr 8.754500e-02 3.087926e-02 2.83507403 4.581507e-03
## body_fat 2.060372e-01 3.637317e-02 5.66453671 1.474223e-08
## Glucose -2.285264e-03 2.915824e-02 -0.07837457 9.375301e-01
## HDL_cholesterol -1.158590e-01 3.009070e-02 -3.85032508 1.179612e-04
## Total_cholesterol 6.891861e-02 2.872491e-02 2.39926285 1.642812e-02
## Sodium -1.061848e-01 3.002554e-02 -3.53648471 4.054900e-04
## Potassium 6.682508e-02 2.630567e-02 2.54032960 1.107480e-02
## Urea -1.485310e-01 3.507104e-02 -4.23514798 2.284012e-05
## Creatinine 1.712076e-02 3.389664e-02 0.50508710 6.134977e-01
## Creat_mgdl 1.200295e-02 3.380294e-02 0.35508596 7.225252e-01
## pack_years 1.554327e-01 2.693205e-02 5.77129284 7.866563e-09
## smoke 5.565307e-01 6.713776e-02 8.28938372 1.138367e-16

### Rscript for MS calculation

#### Read in target sample DNAm data

#### Read in participant list for which to calculate MS

#### Read in CpGs and weights derived from training data (here, these represent CpGs and weights from previous trait EWAS)

a = which(colnames(data) %in% part_ids$ID) meth = data[,a] rm(data) dat = meth rm(meth)

meth = t(dat) rm(dat) meth1 = as.data.frame(meth) meth1$id = as.character(rownames(meth1))

a = which(names(meth1) %in% coef$coef.name)meth2=meth1[,a]meth3=t(meth2)probes<-intersect(coef$coef.name, rownames(meth3)) rownames(coef) = coef$coef.name

b = meth3[probes,] p = coef[probes,]

for (i in probes) { b[i,]= b[i,]*p[i,“coef.value”] }

MS=colSums(b) + coef[1,2] pred_MS = as.data.frame(MS) pred_MS$ID = rownames(pred_MS)

MS_final = merge(part_ids, pred_MS, by=“ID”)

#### Save final file, should contain IDs, phenotype (if included in original participant ID file), and MS

### Regression analyses - MS and MDD

The following script is demonstrated for the simple model, where only age and sex are included as covariates. Scripts are the same for all subsequent models (i.e. phenotype+MS model, lifestyle+phenotype+MS mode).

# Scale continuous variables:
lifestyle_MS[,c(7,8,10,11,13:32)]=scale(lifestyle_MS[,c(7,8,10,11,13:32)])

n <-32 # Change according to number of lifestyle variables to include
glm_mdd_MS <- lapply(14:n, function(x) glm(dep_status ~ age+sex+lifestyle_MS[,x],data=lifestyle_MS,family="binomial"))
summary_glm_mdd_MS <- lapply(glm_mdd_MS, summary)

stats_glm_mdd_MS = lapply(summary_glm_mdd_MS, function(x) x$coefficients[c(4),])
names(stats_glm_mdd_MS) = colnames(lifestyle_MS)[14:32] # Change according to scores
stats_dataframe_MS = data.frame(stats_glm_mdd_MS)

stats_dataframe_MS = t(stats_dataframe_MS)
stats_dataframe_MS = data.frame(stats_dataframe_MS)
print(stats_dataframe_MS)

## Estimate Std..Error z.value Pr...z..
## hdl_chol_MS -0.11287942 0.02740728 -4.118592 3.811942e-05
## total_chol_MS -0.07673225 0.02749613 -2.790657 5.260125e-03
## smoke_m_MS 0.09308468 0.02717398 3.425508 6.136503e-04
## bmi_MS 0.13827863 0.02723483 5.077271 3.828947e-07
## education_MS -0.14203776 0.02664366 -5.331014 9.766565e-08
## educ_MS_0.01 -0.14780651 0.02744805 -5.384956 7.246249e-08
## educ_MS_0.05 -0.12466204 0.02760494 -4.515932 6.303885e-06
## educ_MS_0.1 -0.11897178 0.02762251 -4.307060 1.654389e-05
## educ_MS_0.5 -0.10940424 0.02759900 -3.964066 7.368393e-05
## smoke_j_MS 0.15983353 0.02691832 5.937722 2.890093e-09
## smoke_MS_0.01 0.15928181 0.02707226 5.883580 4.014870e-09
## smoke_MS_0.05 0.15667121 0.02711714 5.777571 7.578675e-09
## smoke_MS_0.1 0.15488237 0.02713121 5.708643 1.138804e-08
## smoke_MS_0.5 0.14816446 0.02714893 5.457469 4.829698e-08
## alcohol_MS 0.06132531 0.02737903 2.239864 2.509976e-02
## alc_MS_0.01 -0.06163363 0.02840317 -2.169956 3.001018e-02
## alc_MS_0.05 -0.08290076 0.02842580 -2.916391 3.541059e-03
## alc_MS_0.1 -0.08913273 0.02840593 -3.137821 1.702088e-03
## alc_MS_0.5 -0.09716984 0.02834031 -3.428679 6.065258e-04

### Co-localization analysis

Colocalization analysis was used to identify colocalized SNPs between MDD and DNA methylation. Howard et al.’s (2019) MDD GWAS was used, where we selected the 102 SNPs associated with MDD as well as SNPs +/- 1Mb, totalling 102 genomic regions. We used GoDMC for DNAm-SNP associations.

R package “gwasglue” was used to query regions +/- 1Mb from the MDD GWAS lead SNP, e.g.:

mdd_positions=variants_chrpos(c(“1:7489302-9489302”, “1:36192741-38192741”, “1:36709328-38709328”))

These variants were then merged with summary statistics from the MDD GWAS and GoDMC for colocalization analysis.

## New names:
## * `Odds Ratio` -> `Odds Ratio...10`
## * `Lower 95% Confidence Interval` -> `Lower 95% Confidence Interval...11`
## * `Upper 95% Confidence Interval` -> `Upper 95% Confidence Interval...12`
## * `Log(Odds Ratio)` -> `Log(Odds Ratio)...13`
## * `Standard error of the Log(Odds Ratio)` -> `Standard error of the Log(Odds Ratio)...14`
## * ...

# MDD - SELECT MAF, BETA, VARBETA
dataset1 <- lapply(godmc_list, `[`, c(1,4,8,42)) # This selects all rows and columns needed for coloc.abf
n <-102 # Change according to number of elements in list
dataset1list <- lapply(1:n, function(x) list(beta=dataset1[[x]]$Effect,varbeta=dataset1[[x]]$varbeta_mdd,MAF=dataset1[[x]]$Freq1,type="cc",s=0.31,N=800607))
names(dataset1list)=names(dataset1)

# GoDMC - SELECT MAF, BETA, VARBETA
dataset2 <- lapply(godmc_list, `[`, c(1,4,15,43)) # This selects all rows and columns needed for coloc.abf
n <-102 # Change according to number of lifestyle variables to include
dataset2list <- lapply(1:n, function(x) list(beta=dataset2[[x]]$beta_a1,varbeta=dataset2[[x]]$varbeta_godmc,type="quant",N=27750))
names(dataset2list)=names(dataset2)

n <-102 # Change according to number of coloc tests to run
coloc_results <- lapply(1:n, function(x) coloc.abf(dataset1list[[x]],dataset2list[[x]], MAF=dataset1list[[x]]$MAF))

## Warning in sdY.est(d$varbeta, d$MAF, d$N): estimating sdY from maf and varbeta,
## please directly supply sdY if known

## PP.H0.abf PP.H1.abf PP.H2.abf PP.H3.abf PP.H4.abf
## 0.00e+00 0.00e+00 2.45e-07 1.00e+00 6.67e-10
## [1] "PP abf for shared variant: 6.67e-08%"

## Warning in sdY.est(d$varbeta, d$MAF, d$N): estimating sdY from maf and varbeta,
## please directly supply sdY if known

## PP.H0.abf PP.H1.abf PP.H2.abf PP.H3.abf PP.H4.abf
## 0.00e+00 0.00e+00 9.22e-07 1.00e+00 2.96e-09
## [1] "PP abf for shared variant: 2.96e-07%"

## Warning in sdY.est(d$varbeta, d$MAF, d$N): estimating sdY from maf and varbeta,
## please directly supply sdY if known

## PP.H0.abf PP.H1.abf PP.H2.abf PP.H3.abf PP.H4.abf
## 0.000000 0.000000 0.046300 0.954000 0.000135
## [1] "PP abf for shared variant: 0.0135%"

## Warning in sdY.est(d$varbeta, d$MAF, d$N): estimating sdY from maf and varbeta,
## please directly supply sdY if known

## PP.H0.abf PP.H1.abf PP.H2.abf PP.H3.abf PP.H4.abf
## 0.00e+00 0.00e+00 1.76e-07 1.00e+00 9.78e-10
## [1] "PP abf for shared variant: 9.78e-08%"

## Warning in sdY.est(d$varbeta, d$MAF, d$N): estimating sdY from maf and varbeta,
## please directly supply sdY if known

## PP.H0.abf PP.H1.abf PP.H2.abf PP.H3.abf PP.H4.abf
## 0.00e+00 0.00e+00 4.88e-06 1.00e+00 1.13e-08
## [1] "PP abf for shared variant: 1.13e-06%"

## Warning in sdY.est(d$varbeta, d$MAF, d$N): estimating sdY from maf and varbeta,
## please directly supply sdY if known

## PP.H0.abf PP.H1.abf PP.H2.abf PP.H3.abf PP.H4.abf
## 0.00e+00 0.00e+00 4.88e-06 1.00e+00 1.13e-08
## [1] "PP abf for shared variant: 1.13e-06%"

## Warning in sdY.est(d$varbeta, d$MAF, d$N): estimating sdY from maf and varbeta,
## please directly supply sdY if known

## PP.H0.abf PP.H1.abf PP.H2.abf PP.H3.abf PP.H4.abf
## 0.00e+00 0.00e+00 2.12e-04 1.00e+00 1.93e-05
## [1] "PP abf for shared variant: 0.00193%"

## Warning in sdY.est(d$varbeta, d$MAF, d$N): estimating sdY from maf and varbeta,
## please directly supply sdY if known

## PP.H0.abf PP.H1.abf PP.H2.abf PP.H3.abf PP.H4.abf
## 0.00e+00 0.00e+00 3.89e-06 1.00e+00 1.37e-08
## [1] "PP abf for shared variant: 1.37e-06%"

## Warning in sdY.est(d$varbeta, d$MAF, d$N): estimating sdY from maf and varbeta,
## please directly supply sdY if known

## PP.H0.abf PP.H1.abf PP.H2.abf PP.H3.abf PP.H4.abf
## 0.00e+00 0.00e+00 1.07e-03 9.99e-01 3.63e-06
## [1] "PP abf for shared variant: 0.000363%"

## Warning in sdY.est(d$varbeta, d$MAF, d$N): estimating sdY from maf and varbeta,
## please directly supply sdY if known

## PP.H0.abf PP.H1.abf PP.H2.abf PP.H3.abf PP.H4.abf
## 0.00e+00 0.00e+00 1.83e-18 1.00e+00 2.17e-20
## [1] "PP abf for shared variant: 2.17e-18%"

## Warning in sdY.est(d$varbeta, d$MAF, d$N): estimating sdY from maf and varbeta,
## please directly supply sdY if known

## PP.H0.abf PP.H1.abf PP.H2.abf PP.H3.abf PP.H4.abf
## 0.00e+00 0.00e+00 1.83e-18 1.00e+00 2.17e-20
## [1] "PP abf for shared variant: 2.17e-18%"

## Warning in sdY.est(d$varbeta, d$MAF, d$N): estimating sdY from maf and varbeta,
## please directly supply sdY if known

## PP.H0.abf PP.H1.abf PP.H2.abf PP.H3.abf PP.H4.abf
## 0.00e+00 0.00e+00 4.83e-07 1.00e+00 9.80e-10
## [1] "PP abf for shared variant: 9.8e-08%"

## Warning in sdY.est(d$varbeta, d$MAF, d$N): estimating sdY from maf and varbeta,
## please directly supply sdY if known

## PP.H0.abf PP.H1.abf PP.H2.abf PP.H3.abf PP.H4.abf
## 0.00e+00 0.00e+00 2.38e-03 9.98e-01 4.48e-06
## [1] "PP abf for shared variant: 0.000448%"

## Warning in sdY.est(d$varbeta, d$MAF, d$N): estimating sdY from maf and varbeta,
## please directly supply sdY if known

## PP.H0.abf PP.H1.abf PP.H2.abf PP.H3.abf PP.H4.abf
## 0.000000 0.000000 0.031400 0.968000 0.000406
## [1] "PP abf for shared variant: 0.0406%"

## Warning in sdY.est(d$varbeta, d$MAF, d$N): estimating sdY from maf and varbeta,
## please directly supply sdY if known

## PP.H0.abf PP.H1.abf PP.H2.abf PP.H3.abf PP.H4.abf
## 0.00e+00 0.00e+00 8.81e-07 1.00e+00 1.69e-07
## [1] "PP abf for shared variant: 1.69e-05%"

## Warning in sdY.est(d$varbeta, d$MAF, d$N): estimating sdY from maf and varbeta,
## please directly supply sdY if known

## PP.H0.abf PP.H1.abf PP.H2.abf PP.H3.abf PP.H4.abf
## 0.00e+00 0.00e+00 5.21e-03 9.95e-01 1.23e-05
## [1] "PP abf for shared variant: 0.00123%"

## Warning in sdY.est(d$varbeta, d$MAF, d$N): estimating sdY from maf and varbeta,
## please directly supply sdY if known

## PP.H0.abf PP.H1.abf PP.H2.abf PP.H3.abf PP.H4.abf
## 0.00e+00 0.00e+00 1.40e-03 9.99e-01 1.31e-05
## [1] "PP abf for shared variant: 0.00131%"

## Warning in sdY.est(d$varbeta, d$MAF, d$N): estimating sdY from maf and varbeta,
## please directly supply sdY if known

## PP.H0.abf PP.H1.abf PP.H2.abf PP.H3.abf PP.H4.abf
## 0.00e+00 0.00e+00 2.63e-05 1.00e+00 7.68e-07
## [1] "PP abf for shared variant: 7.68e-05%"

## Warning in sdY.est(d$varbeta, d$MAF, d$N): estimating sdY from maf and varbeta,
## please directly supply sdY if known

## PP.H0.abf PP.H1.abf PP.H2.abf PP.H3.abf PP.H4.abf
## 0.00e+00 0.00e+00 2.92e-04 1.00e+00 2.01e-06
## [1] "PP abf for shared variant: 0.000201%"

## Warning in sdY.est(d$varbeta, d$MAF, d$N): estimating sdY from maf and varbeta,
## please directly supply sdY if known

## PP.H0.abf PP.H1.abf PP.H2.abf PP.H3.abf PP.H4.abf
## 0.00e+00 0.00e+00 7.06e-05 1.00e+00 3.23e-07
## [1] "PP abf for shared variant: 3.23e-05%"

## Warning in sdY.est(d$varbeta, d$MAF, d$N): estimating sdY from maf and varbeta,
## please directly supply sdY if known

## PP.H0.abf PP.H1.abf PP.H2.abf PP.H3.abf PP.H4.abf
## 0.00e+00 0.00e+00 9.36e-05 1.00e+00 2.48e-07
## [1] "PP abf for shared variant: 2.48e-05%"

## Warning in sdY.est(d$varbeta, d$MAF, d$N): estimating sdY from maf and varbeta,
## please directly supply sdY if known

## PP.H0.abf PP.H1.abf PP.H2.abf PP.H3.abf PP.H4.abf
## 0.00e+00 0.00e+00 5.56e-04 9.99e-01 3.35e-06
## [1] "PP abf for shared variant: 0.000335%"

## Warning in sdY.est(d$varbeta, d$MAF, d$N): estimating sdY from maf and varbeta,
## please directly supply sdY if known

## PP.H0.abf PP.H1.abf PP.H2.abf PP.H3.abf PP.H4.abf
## 0.00e+00 0.00e+00 2.83e-09 1.00e+00 5.30e-12
## [1] "PP abf for shared variant: 5.3e-10%"

## Warning in sdY.est(d$varbeta, d$MAF, d$N): estimating sdY from maf and varbeta,
## please directly supply sdY if known

## PP.H0.abf PP.H1.abf PP.H2.abf PP.H3.abf PP.H4.abf
## 0.00e+00 0.00e+00 3.08e-03 9.97e-01 7.40e-06
## [1] "PP abf for shared variant: 0.00074%"

## Warning in sdY.est(d$varbeta, d$MAF, d$N): estimating sdY from maf and varbeta,
## please directly supply sdY if known

## PP.H0.abf PP.H1.abf PP.H2.abf PP.H3.abf PP.H4.abf
## 0.00e+00 0.00e+00 3.98e-06 1.00e+00 7.18e-09
## [1] "PP abf for shared variant: 7.18e-07%"

## Warning in sdY.est(d$varbeta, d$MAF, d$N): estimating sdY from maf and varbeta,
## please directly supply sdY if known

## PP.H0.abf PP.H1.abf PP.H2.abf PP.H3.abf PP.H4.abf
## 0.0000 0.0000 0.0740 0.9260 0.0003
## [1] "PP abf for shared variant: 0.03%"

## Warning in sdY.est(d$varbeta, d$MAF, d$N): estimating sdY from maf and varbeta,
## please directly supply sdY if known

## PP.H0.abf PP.H1.abf PP.H2.abf PP.H3.abf PP.H4.abf
## 0.00e+00 0.00e+00 2.83e-04 1.00e+00 1.43e-06
## [1] "PP abf for shared variant: 0.000143%"

## Warning in sdY.est(d$varbeta, d$MAF, d$N): estimating sdY from maf and varbeta,
## please directly supply sdY if known

## PP.H0.abf PP.H1.abf PP.H2.abf PP.H3.abf PP.H4.abf
## 0.00e+00 0.00e+00 1.62e-04 1.00e+00 5.15e-07
## [1] "PP abf for shared variant: 5.15e-05%"

## Warning in sdY.est(d$varbeta, d$MAF, d$N): estimating sdY from maf and varbeta,
## please directly supply sdY if known

## PP.H0.abf PP.H1.abf PP.H2.abf PP.H3.abf PP.H4.abf
## 0.00e+00 0.00e+00 2.73e-11 1.00e+00 5.29e-14
## [1] "PP abf for shared variant: 5.29e-12%"

## Warning in sdY.est(d$varbeta, d$MAF, d$N): estimating sdY from maf and varbeta,
## please directly supply sdY if known

## PP.H0.abf PP.H1.abf PP.H2.abf PP.H3.abf PP.H4.abf
## 0.00e+00 0.00e+00 5.31e-04 9.99e-01 6.57e-06
## [1] "PP abf for shared variant: 0.000657%"

## Warning in sdY.est(d$varbeta, d$MAF, d$N): estimating sdY from maf and varbeta,
## please directly supply sdY if known

## PP.H0.abf PP.H1.abf PP.H2.abf PP.H3.abf PP.H4.abf
## 0.000000 0.000000 0.005650 0.994000 0.000034
## [1] "PP abf for shared variant: 0.0034%"

## Warning in sdY.est(d$varbeta, d$MAF, d$N): estimating sdY from maf and varbeta,
## please directly supply sdY if known

## PP.H0.abf PP.H1.abf PP.H2.abf PP.H3.abf PP.H4.abf
## 0.00e+00 0.00e+00 1.48e-04 1.00e+00 3.64e-07
## [1] "PP abf for shared variant: 3.64e-05%"

## Warning in sdY.est(d$varbeta, d$MAF, d$N): estimating sdY from maf and varbeta,
## please directly supply sdY if known

## PP.H0.abf PP.H1.abf PP.H2.abf PP.H3.abf PP.H4.abf
## 0.00e+00 0.00e+00 8.33e-10 1.00e+00 9.52e-12
## [1] "PP abf for shared variant: 9.52e-10%"

## Warning in sdY.est(d$varbeta, d$MAF, d$N): estimating sdY from maf and varbeta,
## please directly supply sdY if known

## PP.H0.abf PP.H1.abf PP.H2.abf PP.H3.abf PP.H4.abf
## 0.00e+00 0.00e+00 1.34e-08 1.00e+00 7.23e-09
## [1] "PP abf for shared variant: 7.23e-07%"

## Warning in sdY.est(d$varbeta, d$MAF, d$N): estimating sdY from maf and varbeta,
## please directly supply sdY if known

## PP.H0.abf PP.H1.abf PP.H2.abf PP.H3.abf PP.H4.abf
## 0.00e+00 0.00e+00 4.52e-05 1.00e+00 3.02e-07
## [1] "PP abf for shared variant: 3.02e-05%"

## Warning in sdY.est(d$varbeta, d$MAF, d$N): estimating sdY from maf and varbeta,
## please directly supply sdY if known

## PP.H0.abf PP.H1.abf PP.H2.abf PP.H3.abf PP.H4.abf
## 0.00e+00 0.00e+00 5.02e-08 1.00e+00 2.07e-09
## [1] "PP abf for shared variant: 2.07e-07%"

## Warning in sdY.est(d$varbeta, d$MAF, d$N): estimating sdY from maf and varbeta,
## please directly supply sdY if known

## PP.H0.abf PP.H1.abf PP.H2.abf PP.H3.abf PP.H4.abf
## 0.00e+00 0.00e+00 3.44e-03 9.97e-01 6.60e-06
## [1] "PP abf for shared variant: 0.00066%"

## Warning in sdY.est(d$varbeta, d$MAF, d$N): estimating sdY from maf and varbeta,
## please directly supply sdY if known

## PP.H0.abf PP.H1.abf PP.H2.abf PP.H3.abf PP.H4.abf
## 0.000000 0.000000 0.000618 0.999000 0.000058
## [1] "PP abf for shared variant: 0.0058%"

## Warning in sdY.est(d$varbeta, d$MAF, d$N): estimating sdY from maf and varbeta,
## please directly supply sdY if known

## PP.H0.abf PP.H1.abf PP.H2.abf PP.H3.abf PP.H4.abf
## 0.00e+00 0.00e+00 1.28e-03 9.99e-01 6.06e-06
## [1] "PP abf for shared variant: 0.000606%"

## Warning in sdY.est(d$varbeta, d$MAF, d$N): estimating sdY from maf and varbeta,
## please directly supply sdY if known

## PP.H0.abf PP.H1.abf PP.H2.abf PP.H3.abf PP.H4.abf
## 0.00e+00 0.00e+00 1.29e-03 9.99e-01 5.65e-05
## [1] "PP abf for shared variant: 0.00565%"

## Warning in sdY.est(d$varbeta, d$MAF, d$N): estimating sdY from maf and varbeta,
## please directly supply sdY if known

## PP.H0.abf PP.H1.abf PP.H2.abf PP.H3.abf PP.H4.abf
## 0.00e+00 0.00e+00 9.32e-04 9.99e-01 1.96e-06
## [1] "PP abf for shared variant: 0.000196%"

## Warning in sdY.est(d$varbeta, d$MAF, d$N): estimating sdY from maf and varbeta,
## please directly supply sdY if known

## PP.H0.abf PP.H1.abf PP.H2.abf PP.H3.abf PP.H4.abf
## 0.00e+00 0.00e+00 2.65e-02 9.73e-01 5.56e-05
## [1] "PP abf for shared variant: 0.00556%"

## Warning in sdY.est(d$varbeta, d$MAF, d$N): estimating sdY from maf and varbeta,
## please directly supply sdY if known

## PP.H0.abf PP.H1.abf PP.H2.abf PP.H3.abf PP.H4.abf
## 0.00e+00 0.00e+00 5.39e-04 9.99e-01 9.98e-07
## [1] "PP abf for shared variant: 9.98e-05%"

## Warning in sdY.est(d$varbeta, d$MAF, d$N): estimating sdY from maf and varbeta,
## please directly supply sdY if known

## PP.H0.abf PP.H1.abf PP.H2.abf PP.H3.abf PP.H4.abf
## 0.00e+00 0.00e+00 2.22e-06 1.00e+00 5.17e-09
## [1] "PP abf for shared variant: 5.17e-07%"

## Warning in sdY.est(d$varbeta, d$MAF, d$N): estimating sdY from maf and varbeta,
## please directly supply sdY if known

## PP.H0.abf PP.H1.abf PP.H2.abf PP.H3.abf PP.H4.abf
## 0.00e+00 0.00e+00 2.22e-06 1.00e+00 5.17e-09
## [1] "PP abf for shared variant: 5.17e-07%"

## Warning in sdY.est(d$varbeta, d$MAF, d$N): estimating sdY from maf and varbeta,
## please directly supply sdY if known

## PP.H0.abf PP.H1.abf PP.H2.abf PP.H3.abf PP.H4.abf
## 0.00e+00 0.00e+00 2.22e-06 1.00e+00 5.17e-09
## [1] "PP abf for shared variant: 5.17e-07%"

## Warning in sdY.est(d$varbeta, d$MAF, d$N): estimating sdY from maf and varbeta,
## please directly supply sdY if known

## PP.H0.abf PP.H1.abf PP.H2.abf PP.H3.abf PP.H4.abf
## 0.00e+00 0.00e+00 1.14e-04 1.00e+00 6.30e-07
## [1] "PP abf for shared variant: 6.3e-05%"

## Warning in sdY.est(d$varbeta, d$MAF, d$N): estimating sdY from maf and varbeta,
## please directly supply sdY if known

## PP.H0.abf PP.H1.abf PP.H2.abf PP.H3.abf PP.H4.abf
## 0.0000 0.0000 0.4580 0.5410 0.0012
## [1] "PP abf for shared variant: 0.12%"

## Warning in sdY.est(d$varbeta, d$MAF, d$N): estimating sdY from maf and varbeta,
## please directly supply sdY if known

## PP.H0.abf PP.H1.abf PP.H2.abf PP.H3.abf PP.H4.abf
## 0.00e+00 0.00e+00 1.87e-04 1.00e+00 3.52e-07
## [1] "PP abf for shared variant: 3.52e-05%"

## Warning in sdY.est(d$varbeta, d$MAF, d$N): estimating sdY from maf and varbeta,
## please directly supply sdY if known

## PP.H0.abf PP.H1.abf PP.H2.abf PP.H3.abf PP.H4.abf
## 0.00e+00 0.00e+00 1.02e-04 1.00e+00 2.78e-07
## [1] "PP abf for shared variant: 2.78e-05%"

## Warning in sdY.est(d$varbeta, d$MAF, d$N): estimating sdY from maf and varbeta,
## please directly supply sdY if known

## PP.H0.abf PP.H1.abf PP.H2.abf PP.H3.abf PP.H4.abf
## 0.00e+00 0.00e+00 6.64e-05 1.00e+00 3.50e-07
## [1] "PP abf for shared variant: 3.5e-05%"

## Warning in sdY.est(d$varbeta, d$MAF, d$N): estimating sdY from maf and varbeta,
## please directly supply sdY if known

## PP.H0.abf PP.H1.abf PP.H2.abf PP.H3.abf PP.H4.abf
## 0.000000 0.000000 0.000291 0.975000 0.024400
## [1] "PP abf for shared variant: 2.44%"

## Warning in sdY.est(d$varbeta, d$MAF, d$N): estimating sdY from maf and varbeta,
## please directly supply sdY if known

## PP.H0.abf PP.H1.abf PP.H2.abf PP.H3.abf PP.H4.abf
## 0.00e+00 0.00e+00 3.31e-04 1.00e+00 2.28e-06
## [1] "PP abf for shared variant: 0.000228%"

## Warning in sdY.est(d$varbeta, d$MAF, d$N): estimating sdY from maf and varbeta,
## please directly supply sdY if known

## PP.H0.abf PP.H1.abf PP.H2.abf PP.H3.abf PP.H4.abf
## 0.00e+00 0.00e+00 2.76e-07 7.26e-01 2.74e-01
## [1] "PP abf for shared variant: 27.4%"

## Warning in sdY.est(d$varbeta, d$MAF, d$N): estimating sdY from maf and varbeta,
## please directly supply sdY if known

## PP.H0.abf PP.H1.abf PP.H2.abf PP.H3.abf PP.H4.abf
## 0.000 0.000 0.001 0.287 0.712
## [1] "PP abf for shared variant: 71.2%"

## Warning in sdY.est(d$varbeta, d$MAF, d$N): estimating sdY from maf and varbeta,
## please directly supply sdY if known

## PP.H0.abf PP.H1.abf PP.H2.abf PP.H3.abf PP.H4.abf
## 0.00e+00 0.00e+00 3.60e-10 1.00e+00 4.58e-12
## [1] "PP abf for shared variant: 4.58e-10%"

## Warning in sdY.est(d$varbeta, d$MAF, d$N): estimating sdY from maf and varbeta,
## please directly supply sdY if known

## PP.H0.abf PP.H1.abf PP.H2.abf PP.H3.abf PP.H4.abf
## 0.00e+00 0.00e+00 6.05e-06 1.00e+00 1.61e-08
## [1] "PP abf for shared variant: 1.61e-06%"

## Warning in sdY.est(d$varbeta, d$MAF, d$N): estimating sdY from maf and varbeta,
## please directly supply sdY if known

## PP.H0.abf PP.H1.abf PP.H2.abf PP.H3.abf PP.H4.abf
## 0.00e+00 0.00e+00 1.11e-04 1.00e+00 1.11e-06
## [1] "PP abf for shared variant: 0.000111%"

## Warning in sdY.est(d$varbeta, d$MAF, d$N): estimating sdY from maf and varbeta,
## please directly supply sdY if known

## PP.H0.abf PP.H1.abf PP.H2.abf PP.H3.abf PP.H4.abf
## 0.00000 0.00000 0.00367 0.99300 0.00382
## [1] "PP abf for shared variant: 0.382%"

## Warning in sdY.est(d$varbeta, d$MAF, d$N): estimating sdY from maf and varbeta,
## please directly supply sdY if known

## PP.H0.abf PP.H1.abf PP.H2.abf PP.H3.abf PP.H4.abf
## 0.00e+00 0.00e+00 5.26e-03 9.95e-01 1.65e-05
## [1] "PP abf for shared variant: 0.00165%"

## Warning in sdY.est(d$varbeta, d$MAF, d$N): estimating sdY from maf and varbeta,
## please directly supply sdY if known

## PP.H0.abf PP.H1.abf PP.H2.abf PP.H3.abf PP.H4.abf
## 0.00e+00 0.00e+00 1.08e-02 9.89e-01 2.17e-05
## [1] "PP abf for shared variant: 0.00217%"

## Warning in sdY.est(d$varbeta, d$MAF, d$N): estimating sdY from maf and varbeta,
## please directly supply sdY if known

## PP.H0.abf PP.H1.abf PP.H2.abf PP.H3.abf PP.H4.abf
## 0.00e+00 0.00e+00 4.28e-04 1.00e+00 2.58e-06
## [1] "PP abf for shared variant: 0.000258%"

## Warning in sdY.est(d$varbeta, d$MAF, d$N): estimating sdY from maf and varbeta,
## please directly supply sdY if known

## PP.H0.abf PP.H1.abf PP.H2.abf PP.H3.abf PP.H4.abf
## 0.00e+00 0.00e+00 3.89e-04 1.00e+00 8.58e-07
## [1] "PP abf for shared variant: 8.58e-05%"

## Warning in sdY.est(d$varbeta, d$MAF, d$N): estimating sdY from maf and varbeta,
## please directly supply sdY if known

## PP.H0.abf PP.H1.abf PP.H2.abf PP.H3.abf PP.H4.abf
## 0.00e+00 0.00e+00 6.02e-11 1.00e+00 1.34e-13
## [1] "PP abf for shared variant: 1.34e-11%"

## Warning in sdY.est(d$varbeta, d$MAF, d$N): estimating sdY from maf and varbeta,
## please directly supply sdY if known

## PP.H0.abf PP.H1.abf PP.H2.abf PP.H3.abf PP.H4.abf
## 0.00e+00 0.00e+00 6.33e-06 1.00e+00 1.19e-07
## [1] "PP abf for shared variant: 1.19e-05%"

## Warning in sdY.est(d$varbeta, d$MAF, d$N): estimating sdY from maf and varbeta,
## please directly supply sdY if known

## PP.H0.abf PP.H1.abf PP.H2.abf PP.H3.abf PP.H4.abf
## 0.000000 0.000000 0.000102 0.992000 0.007960
## [1] "PP abf for shared variant: 0.796%"

## Warning in sdY.est(d$varbeta, d$MAF, d$N): estimating sdY from maf and varbeta,
## please directly supply sdY if known

## PP.H0.abf PP.H1.abf PP.H2.abf PP.H3.abf PP.H4.abf
## 0.00e+00 0.00e+00 3.84e-05 1.00e+00 6.77e-08
## [1] "PP abf for shared variant: 6.77e-06%"

## Warning in sdY.est(d$varbeta, d$MAF, d$N): estimating sdY from maf and varbeta,
## please directly supply sdY if known

## PP.H0.abf PP.H1.abf PP.H2.abf PP.H3.abf PP.H4.abf
## 0.00e+00 0.00e+00 1.38e-04 1.00e+00 8.26e-07
## [1] "PP abf for shared variant: 8.26e-05%"

## Warning in sdY.est(d$varbeta, d$MAF, d$N): estimating sdY from maf and varbeta,
## please directly supply sdY if known

## PP.H0.abf PP.H1.abf PP.H2.abf PP.H3.abf PP.H4.abf
## 0.00e+00 0.00e+00 1.02e-02 9.90e-01 3.14e-05
## [1] "PP abf for shared variant: 0.00314%"

## Warning in sdY.est(d$varbeta, d$MAF, d$N): estimating sdY from maf and varbeta,
## please directly supply sdY if known

## PP.H0.abf PP.H1.abf PP.H2.abf PP.H3.abf PP.H4.abf
## 0.000000 0.000000 0.055100 0.945000 0.000317
## [1] "PP abf for shared variant: 0.0317%"

## Warning in sdY.est(d$varbeta, d$MAF, d$N): estimating sdY from maf and varbeta,
## please directly supply sdY if known

## PP.H0.abf PP.H1.abf PP.H2.abf PP.H3.abf PP.H4.abf
## 0.00e+00 0.00e+00 1.45e-16 1.00e+00 1.35e-09
## [1] "PP abf for shared variant: 1.35e-07%"

## Warning in sdY.est(d$varbeta, d$MAF, d$N): estimating sdY from maf and varbeta,
## please directly supply sdY if known

## PP.H0.abf PP.H1.abf PP.H2.abf PP.H3.abf PP.H4.abf
## 0.00e+00 0.00e+00 1.07e-04 1.00e+00 3.27e-07
## [1] "PP abf for shared variant: 3.27e-05%"

## Warning in sdY.est(d$varbeta, d$MAF, d$N): estimating sdY from maf and varbeta,
## please directly supply sdY if known

## PP.H0.abf PP.H1.abf PP.H2.abf PP.H3.abf PP.H4.abf
## 0.00e+00 0.00e+00 1.77e-05 1.00e+00 1.80e-07
## [1] "PP abf for shared variant: 1.8e-05%"

## Warning in sdY.est(d$varbeta, d$MAF, d$N): estimating sdY from maf and varbeta,
## please directly supply sdY if known

## PP.H0.abf PP.H1.abf PP.H2.abf PP.H3.abf PP.H4.abf
## 0.00e+00 0.00e+00 4.55e-06 1.00e+00 8.20e-09
## [1] "PP abf for shared variant: 8.2e-07%"

## Warning in sdY.est(d$varbeta, d$MAF, d$N): estimating sdY from maf and varbeta,
## please directly supply sdY if known

## PP.H0.abf PP.H1.abf PP.H2.abf PP.H3.abf PP.H4.abf
## 0.00e+00 0.00e+00 8.31e-06 1.00e+00 2.41e-08
## [1] "PP abf for shared variant: 2.41e-06%"

## Warning in sdY.est(d$varbeta, d$MAF, d$N): estimating sdY from maf and varbeta,
## please directly supply sdY if known

## PP.H0.abf PP.H1.abf PP.H2.abf PP.H3.abf PP.H4.abf
## 0.00e+00 0.00e+00 8.83e-04 9.99e-01 2.76e-06
## [1] "PP abf for shared variant: 0.000276%"

## Warning in sdY.est(d$varbeta, d$MAF, d$N): estimating sdY from maf and varbeta,
## please directly supply sdY if known

## PP.H0.abf PP.H1.abf PP.H2.abf PP.H3.abf PP.H4.abf
## 0.00e+00 0.00e+00 8.84e-04 9.99e-01 2.76e-06
## [1] "PP abf for shared variant: 0.000276%"

## Warning in sdY.est(d$varbeta, d$MAF, d$N): estimating sdY from maf and varbeta,
## please directly supply sdY if known

## PP.H0.abf PP.H1.abf PP.H2.abf PP.H3.abf PP.H4.abf
## 0.0000 0.0000 0.0385 0.9480 0.0134
## [1] "PP abf for shared variant: 1.34%"

## Warning in sdY.est(d$varbeta, d$MAF, d$N): estimating sdY from maf and varbeta,
## please directly supply sdY if known

## PP.H0.abf PP.H1.abf PP.H2.abf PP.H3.abf PP.H4.abf
## 0.00e+00 0.00e+00 6.29e-06 1.00e+00 2.13e-08
## [1] "PP abf for shared variant: 2.13e-06%"

## Warning in sdY.est(d$varbeta, d$MAF, d$N): estimating sdY from maf and varbeta,
## please directly supply sdY if known

## PP.H0.abf PP.H1.abf PP.H2.abf PP.H3.abf PP.H4.abf
## 0.00e+00 0.00e+00 1.13e-05 1.00e+00 2.16e-06
## [1] "PP abf for shared variant: 0.000216%"

## Warning in sdY.est(d$varbeta, d$MAF, d$N): estimating sdY from maf and varbeta,
## please directly supply sdY if known

## PP.H0.abf PP.H1.abf PP.H2.abf PP.H3.abf PP.H4.abf
## 0.00e+00 0.00e+00 1.13e-05 1.00e+00 2.16e-06
## [1] "PP abf for shared variant: 0.000216%"

## Warning in sdY.est(d$varbeta, d$MAF, d$N): estimating sdY from maf and varbeta,
## please directly supply sdY if known

## PP.H0.abf PP.H1.abf PP.H2.abf PP.H3.abf PP.H4.abf
## 0.00e+00 0.00e+00 9.23e-05 9.98e-01 1.98e-03
## [1] "PP abf for shared variant: 0.198%"

## Warning in sdY.est(d$varbeta, d$MAF, d$N): estimating sdY from maf and varbeta,
## please directly supply sdY if known

## PP.H0.abf PP.H1.abf PP.H2.abf PP.H3.abf PP.H4.abf
## 0.00e+00 0.00e+00 2.58e-04 1.00e+00 1.49e-06
## [1] "PP abf for shared variant: 0.000149%"

## Warning in sdY.est(d$varbeta, d$MAF, d$N): estimating sdY from maf and varbeta,
## please directly supply sdY if known

## PP.H0.abf PP.H1.abf PP.H2.abf PP.H3.abf PP.H4.abf
## 0.00000 0.00000 0.99600 0.00170 0.00182
## [1] "PP abf for shared variant: 0.182%"

## Warning in sdY.est(d$varbeta, d$MAF, d$N): estimating sdY from maf and varbeta,
## please directly supply sdY if known

## PP.H0.abf PP.H1.abf PP.H2.abf PP.H3.abf PP.H4.abf
## 0.0e+00 0.0e+00 4.9e-07 1.0e+00 1.2e-09
## [1] "PP abf for shared variant: 1.2e-07%"

## Warning in sdY.est(d$varbeta, d$MAF, d$N): estimating sdY from maf and varbeta,
## please directly supply sdY if known

## PP.H0.abf PP.H1.abf PP.H2.abf PP.H3.abf PP.H4.abf
## 0.00e+00 0.00e+00 4.74e-09 1.00e+00 8.79e-12
## [1] "PP abf for shared variant: 8.79e-10%"

## Warning in sdY.est(d$varbeta, d$MAF, d$N): estimating sdY from maf and varbeta,
## please directly supply sdY if known

## PP.H0.abf PP.H1.abf PP.H2.abf PP.H3.abf PP.H4.abf
## 0.00e+00 0.00e+00 9.95e-04 9.99e-01 5.25e-06
## [1] "PP abf for shared variant: 0.000525%"

## Warning in sdY.est(d$varbeta, d$MAF, d$N): estimating sdY from maf and varbeta,
## please directly supply sdY if known

## PP.H0.abf PP.H1.abf PP.H2.abf PP.H3.abf PP.H4.abf
## 0.00e+00 0.00e+00 3.03e-04 1.00e+00 5.47e-07
## [1] "PP abf for shared variant: 5.47e-05%"

## Warning in sdY.est(d$varbeta, d$MAF, d$N): estimating sdY from maf and varbeta,
## please directly supply sdY if known

## PP.H0.abf PP.H1.abf PP.H2.abf PP.H3.abf PP.H4.abf
## 0.00e+00 0.00e+00 5.44e-10 9.98e-01 1.69e-03
## [1] "PP abf for shared variant: 0.169%"

## Warning in sdY.est(d$varbeta, d$MAF, d$N): estimating sdY from maf and varbeta,
## please directly supply sdY if known

## PP.H0.abf PP.H1.abf PP.H2.abf PP.H3.abf PP.H4.abf
## 0.00e+00 0.00e+00 3.96e-11 1.00e+00 3.52e-12
## [1] "PP abf for shared variant: 3.52e-10%"

## Warning in sdY.est(d$varbeta, d$MAF, d$N): estimating sdY from maf and varbeta,
## please directly supply sdY if known

## PP.H0.abf PP.H1.abf PP.H2.abf PP.H3.abf PP.H4.abf
## 0.00e+00 0.00e+00 6.86e-03 9.93e-01 2.27e-05
## [1] "PP abf for shared variant: 0.00227%"

## Warning in sdY.est(d$varbeta, d$MAF, d$N): estimating sdY from maf and varbeta,
## please directly supply sdY if known

## PP.H0.abf PP.H1.abf PP.H2.abf PP.H3.abf PP.H4.abf
## 0.00e+00 0.00e+00 2.07e-02 9.79e-01 8.95e-05
## [1] "PP abf for shared variant: 0.00895%"

## Warning in sdY.est(d$varbeta, d$MAF, d$N): estimating sdY from maf and varbeta,
## please directly supply sdY if known

## PP.H0.abf PP.H1.abf PP.H2.abf PP.H3.abf PP.H4.abf
## 0.00e+00 0.00e+00 1.48e-04 1.00e+00 8.67e-06
## [1] "PP abf for shared variant: 0.000867%"

## Warning in sdY.est(d$varbeta, d$MAF, d$N): estimating sdY from maf and varbeta,
## please directly supply sdY if known

## PP.H0.abf PP.H1.abf PP.H2.abf PP.H3.abf PP.H4.abf
## 0.00000 0.00000 0.89400 0.10400 0.00173
## [1] "PP abf for shared variant: 0.173%"

## Warning in sdY.est(d$varbeta, d$MAF, d$N): estimating sdY from maf and varbeta,
## please directly supply sdY if known

## PP.H0.abf PP.H1.abf PP.H2.abf PP.H3.abf PP.H4.abf
## 0.00e+00 0.00e+00 8.89e-11 1.00e+00 3.17e-13
## [1] "PP abf for shared variant: 3.17e-11%"

## Warning in sdY.est(d$varbeta, d$MAF, d$N): estimating sdY from maf and varbeta,
## please directly supply sdY if known

## PP.H0.abf PP.H1.abf PP.H2.abf PP.H3.abf PP.H4.abf
## 0.00e+00 0.00e+00 5.94e-04 9.99e-01 1.25e-06
## [1] "PP abf for shared variant: 0.000125%"

## Warning in sdY.est(d$varbeta, d$MAF, d$N): estimating sdY from maf and varbeta,
## please directly supply sdY if known

## PP.H0.abf PP.H1.abf PP.H2.abf PP.H3.abf PP.H4.abf
## 0.00000 0.00000 0.15400 0.84000 0.00587
## [1] "PP abf for shared variant: 0.587%"

## Warning in sdY.est(d$varbeta, d$MAF, d$N): estimating sdY from maf and varbeta,
## please directly supply sdY if known

## PP.H0.abf PP.H1.abf PP.H2.abf PP.H3.abf PP.H4.abf
## 0.00e+00 0.00e+00 9.37e-04 9.99e-01 2.63e-06
## [1] "PP abf for shared variant: 0.000263%"

## Warning in sdY.est(d$varbeta, d$MAF, d$N): estimating sdY from maf and varbeta,
## please directly supply sdY if known

## PP.H0.abf PP.H1.abf PP.H2.abf PP.H3.abf PP.H4.abf
## 0.00e+00 0.00e+00 2.09e-07 1.00e+00 8.94e-08
## [1] "PP abf for shared variant: 8.94e-06%"

## Warning in sdY.est(d$varbeta, d$MAF, d$N): estimating sdY from maf and varbeta,
## please directly supply sdY if known

## PP.H0.abf PP.H1.abf PP.H2.abf PP.H3.abf PP.H4.abf
## 0.00e+00 0.00e+00 3.47e-03 9.97e-01 7.28e-06
## [1] "PP abf for shared variant: 0.000728%"

## Warning in sdY.est(d$varbeta, d$MAF, d$N): estimating sdY from maf and varbeta,
## please directly supply sdY if known

## PP.H0.abf PP.H1.abf PP.H2.abf PP.H3.abf PP.H4.abf
## 0.00e+00 0.00e+00 1.13e-03 9.99e-01 2.01e-06
## [1] "PP abf for shared variant: 0.000201%"

## Warning in sdY.est(d$varbeta, d$MAF, d$N): estimating sdY from maf and varbeta,
## please directly supply sdY if known

## PP.H0.abf PP.H1.abf PP.H2.abf PP.H3.abf PP.H4.abf
## 0.00e+00 0.00e+00 1.60e-03 9.98e-01 5.44e-06
## [1] "PP abf for shared variant: 0.000544%"

names(coloc_results)=names(dataset1list)

## R^2 Plot

The following R^2 plot represents variance explained by each MS in MDD after adjusting for their corresponding phenotypes and lifestyle factors (BMI, alcohol consumption, smoking status, pack years). All other plots were created in the same way. The size of the axis title and text were changed here as the script was too large.

R2=matrix(c(0.05, 0.03,0.03,0.06,0.02,0.02,0.02,0.03,0.01,0.03,0.03,0.03,0.03,0.004,0.07,0.09,0.09,0.09,
 "HDL chol (MW)","Total chol (MW)", "BMI (MW)","Education (MW)","Education (0.01)","Education (0.05)", "Education (0.1)","Education (0.5)",
 "Smoking (MW)","Smoking (0.01)","Smoking (0.05)","Smoking (0.1)","Smoking (0.5)",
 "Alcohol (MW)","Alcohol (0.01)", "Alcohol (0.05)", "Alcohol (0.1)", "Alcohol (0.5)"), nrow=18,ncol=2)
colnames(R2)[1:2]<-c("R2","MRS")
R2=as.data.frame(R2)
R2$R2=as.numeric(levels(R2$R2))[R2$R2]
R2$MRS=factor(R2$MRS,levels=R2$MRS)


rsq_plot<-ggplot(data=R2, aes(x=MRS, y=R2,fill=MRS)) +
 geom_bar(stat="identity") +
 theme_minimal() +
 scale_fill_got_d(option = "Daenerys")+
 ylim(0,0.1)+
 theme(legend.position="none")+
 ggtitle("MRS and MDD") +
 xlab("MRS") + ylab("Variance explained (%)") +
 theme(plot.title = element_text(hjust = 0.5, size = 12, face="bold")) +
 theme(axis.title=element_text(size=12, face = "bold")) +
 theme(axis.text=element_text(size=9)) +
 theme(axis.text.x = element_text(angle = 60,vjust=0.7))
rsq_plot


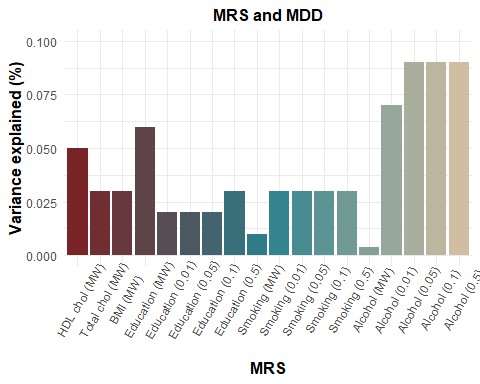

Supplement: Supplementary file 4 [file mmc4.docx]
