# Supplementary material for: Complex trait methylation scores in the prediction of major depressive disorder
Source: eBioMedicine. 2022 Apr 29;79:104000. doi: 10.1016/j.ebiom.2022.104000 (PMC9062752; doi:10.1016/j.ebiom.2022.104000)
Supplement: Supplementary file 5 [file mmc5.docx]

**Complex trait methylation scores in the prediction of major depressive disorder**

Miruna C. Barbu, PhD^1*^, Carmen Amador, PhD^2^, Alex S.F. Kwong, PhD^1^, Xueyi Shen, PhD^1^, Mark J.Adams, PhD^1^, David M. Howard, PhD^1,3^, Rosie M. Walker, PhD^4^, Stewart W. Morris, MSc^4^, Josine L. Min, PhD^5^, Genetics of DNA Methylation Consortium^5^, Chunyu Liu, PhD^6^, Jenny van Dongen, PhD^7^, Mohsen Ghanbari, MD^8^, Caroline Relton, PhD^5^, David J. Porteous, PhD^4^, Archie Campbell, MA^4^, Kathryn L. Evans, PhD^4^, Heather C. Whalley, PhD^1^, Andrew M. McIntosh, MD^1^

^1^Division of Psychiatry, The University of Edinburgh, Royal Edinburgh Hospital, Edinburgh, UK

^2^MRC Human Genetics Unit, The Institute of Genetics and Cancer, The University of Edinburgh, UK

^3^Social, Genetic and Developmental Psychiatry Centre, Institute of Psychiatry, Psychology & Neuroscience, King's College London, UK

^4^Centre for Genomic and Experimental Medicine, The Institute of Genetics and Cancer, The University of Edinburgh, UK

^5^Medical Research Council Integrative Epidemiology Unit, Bristol Medical School, Population Health Sciences, University of Bristol, Bristol, United Kingdom

^6^Department of Biostatistics, Boston University, Boston, MA, USA, The Framingham Heart Study, Framingham, MA, USA

^7^Department of Biological Psychology, Vrije Universiteit Amsterdam, Amsterdam, The Netherlands

^8^Department of Epidemiology, Erasmus University Medical Center Rotterdam, the Netherlands

| Word count (abstract) | 317 |
| --- | --- |
| Word count (main text) | 5,983 |
| Figures | 3 |
| Tables | 6 |
| Supplementary Information | 1 |
| Supplementary Data | 2 |

*Corresponding author: Dr Miruna C Barbu

E-Mail: [mbarbu@ed.ac.uk](mailto:mbarbu@ed.ac.uk)

Address:

University of Edinburgh, Division of Psychiatry, Royal Edinburgh Hospital, Morningside Park

Edinburgh EH10 5HF

United Kingdom

**Abstract**

**Background:** DNA methylation (DNAm) is associated with time-varying environmental factors that contribute to major depressive disorder (MDD) risk. We sought to test whether DNAm signatures of lifestyle and biochemical factors were associated with MDD to reveal dynamic biomarkers of MDD risk that may be amenable to lifestyle interventions.

**Methods:** Here, we calculated methylation scores (MS) at multiple p-value thresholds for lifestyle (BMI, smoking, alcohol consumption, and educational attainment) and biochemical (high-density lipoprotein (HDL) and total cholesterol) factors in Generation Scotland (GS) (N=9,502) and in a replication cohort (ALSPAC_adults_, N=565), using CpG sites reported in previous well-powered methylome-wide association studies. We also compared their predictive accuracy for MDD to a MDD MS in an independent GS sub-sample (N=4,432).

**Findings:** Each trait MS was significantly associated with its corresponding phenotype in GS (β_range_=0.089-1.457) and in ALSPAC (β_range_=0.078-2.533). Each MS was also significantly associated with MDD before and after adjustment for its corresponding phenotype in GS (β_range_=0.053-0.145). After accounting for relevant lifestyle factors, MS for educational attainment (β=0.094) and alcohol consumption (MS_p-value_<0.01-0.5; β_range_=-0.069--0.083) remained significantly associated with MDD in GS. Smoking (AUC=0.569) and educational attainment (AUC=0.585) MSs could discriminate MDD from controls better than the MDD MS (AUC=0.553) in the independent GS sub-sample. Analyses implicating MDD did not replicate across ALSPAC, although the direction of effect was consistent for all traits when adjusting for the MS corresponding phenotypes.

**Interpretation:** We showed that lifestyle and biochemical MS were associated with MDD before and after adjustment for their corresponding phenotypes (p_nominal_<0.05), but not when smoking, alcohol consumption, and BMI were also included as covariates. MDD results did not replicate in the smaller, female-only independent ALSPAC cohort (N_ALSPAC_=565; N_GS_=9,502), potentially due to demographic differences or low statistical power, but effect sizes were consistent with the direction reported in GS. DNAm scores for modifiable MDD risk factors may contribute to disease vulnerability and, in some cases, explain additional variance to their observed phenotypes.

**Funding** Wellcome Trust.

**Keywords**: DNA methylation, methylation score, environmental factors, major depressive disorder, Generation Scotland, ALSPAC

**Introduction**

Major depressive disorder (MDD) is a prevalent psychiatric disorder and is a leading cause of disability worldwide (1). MDD is moderately heritable (h^2^=37%) and is known to result from a complex combination of genetic and environmental risk factors (1). Polygenic risk scores (PRS) derived from large-scale genome-wide association studies (GWAS) explain approximately 1.5-3.2% of MDD risk in independent cohorts (2). In addition, a number of modifiable lifestyle factors are known to associate with MDD, including alcohol intake, smoking, sleeping pattern, diet, and body mass index (BMI) (3,4).

Recently, methylome-wide association studies (MWAS) have begun to identify depressive symptom associations with differential DNA methylation (DNAm) at cytosine-phosphate-guanine (CpG) sites annotated to genes implicated in disorder- and neural-related traits (5,6). Further, methylation scores (MS) for MDD explain additional variance in the disorder when modelled alongside PRS and risk-associated environmental factors, such as smoking, alcohol consumption, and BMI (7,8). However, a large proportion of variance in MDD remains unexplained after accounting for MDD genetic and methylation risk alongside environmental factors.

Recent studies using both methylome-wide association and penalised regression methods have identified DNAm markers for modifiable lifestyle factors, that are measured peripherally in whole blood and can be used for MS estimation (9–13). There are now well-established MWAS for a number of lifestyle factors that are relevant to MDD, including smoking (13), BMI (10), and alcohol consumption (11). In addition, using penalised regression, McCartney et al. (2018) showed that DNAm predictors for complex traits, including BMI, smoking, educational attainment, and total and HDL cholesterol increased the variance explained in these traits when modelled alongside PRS (14). This finding is of interest in the application to multifactorial diseases, where modelling PRS alongside MS for relevant risk factors may enhance prediction. For instance, a recent study showed that a risk model combining lung cancer PRS, a smoking-associated MS, and environmental factors such as pack years predicted lung cancer with a higher accuracy than models including individual scores (AUC_PRS_=0.571, AUC_MS_=0.628, AUC_joint_=0.654), with the increase in AUC being mostly attributable to the MS (15). The study indicates that calculating MS for disease-relevant environmental factors may uncover biomarkers for disease stratification and treatment (15) that may be responsive to lifestyle modifications.

Although several environmental factors with widespread effects on the methylome are known to be associated with MDD, the associations between their epigenetic signatures and MDD has not yet been investigated. Risk prediction models including methylation scores for dynamically changing MDD-associated environmental variables have the potential to increase prediction accuracy for the disorder by capturing an archive of longitudinal exposure. In addition, DNAm biomarkers based on environmental risk factors may lead to the development of novel techniques to measure the efficacy of lifestyle interventions more rapidly, providing potentially useful feedback to both clinicians and patients.

Here, we selected four lifestyle factors (smoking status, alcohol consumption, BMI, educational attainment) and 2 biochemical variables (total cholesterol, high-density lipoprotein (HDL) cholesterol) in N=9,502 individuals in Generation Scotland: the Scottish Family Health Study (GS) that are phenotypically associated with MDD (3,4). We then conducted a literature search to identify well-powered MWAS of these traits.

The aim of the current study was to compute MSs for these MDD-associated risk factors using methylome-wide significant CpGs (9–13). For those variables where full summary statistics were available (alcohol consumption (11), educational attainment (12), smoking status (13)), we additionally calculated MS using four additional p-value thresholds, including p< 0.01, 0.05, 0.1, and 0.5 to investigate whether MSs that include a larger number of CpGs would increase prediction. Associations between the MSs and MDD and their corresponding phenotypes were assessed in 9,502 individuals in GS. We further split GS into a training (N=5,078) and testing (N=4,432) sample to calculate a MDD MS and compared this to complex trait MS in the testing GS sub-sample (N=4,432).

We used an age-matched subset of mothers (mean age=47.96 years, N=565) in the Avon Longitudinal Study of Parents and Children (ALSPAC) cohort to replicate findings in GS. To investigate further concordant signals with MDD, we assessed whether single nucleotide polymorphisms (SNPs) associated with methylation at CpG sites comprising each MS (mQTLs) were colocalised with SNPs associated with MDD.

**Methods**

**Training panels**

To conduct our analyses, we included summary-level data from 6 previous MWASs (educational attainment, HDL cholesterol, total cholesterol, smoking status, alcohol consumption, and BMI (9–13)). For educational attainment, smoking status, and alcohol consumption, full summary statistics were available and obtained directly from the respective authors. For BMI, HDL and total cholesterol, methylome-wide summary statistics were obtained from the EWAS catalog (<http://www.ewascatalog.org/>), after permission was obtained from the EWAS Catalog team (16). Further information regarding each MWAS, including cohort selection, statistical analysis, and demographic information, is available in the Supplementary Table 1 and in each study (9–13).

**Target panels**

*Generation Scotland – Scottish Family Health Study (GS)*

GS is a family-based population cohort aiming to investigate the genetic and environmental causes of health and disease in approximately 24,000 participants aged 18-98 years in Scotland. Baseline data was collected between 2006 and 2011 and includes detailed information on a broad range of variables, including lifestyle and environmental factors, mental health, and medication (17,18). DNA is also available from blood samples taken at the time of recruitment from more than 20,000 consenting participants.

*Avon Longitudinal Study of Parents and Children (ALSPAC)*

ALSPAC is a population-based study in the South-West of England aiming to investigate the effects of multiple factors on health and development. Pregnant women were recruited between April 1991 and December 1992, with the initial number of pregnancies enrolled being 14,541. The cohort now consists of 13,761 mothers, their partners, and their 14,901 children (now young adults) (19–21). The main replication sample in the current study comes from the mothers’ follow-up timepoint (mean age=47.96; see Table 2 for further demographic characteristics) (22). Further information regarding the sample is given in the
Supplemental Materials.

**Ethics**

GS received ethical approval from NHS Tayside Research Ethics Committee (REC reference number 05/S1401/89) and has Research Tissue Bank Status (reference: 20/ES/0021). Written informed consent was obtained from all participants.

ALSPAC received ethical approval from the ALSPAC Ethics and Law Committee and the Local Research Ethics Committees. Written informed consent was obtained from all participants and consent for biological samples has been collected in accordance with the Human Tissue Act (2004). Please note that the study website contains details of all the data that is available through a fully searchable data dictionary and variable search tool: <http://www.bristol.ac.uk/alspac/researchers/our-data/>.

**Phenotypes**

*GS*

MDD status was measured using the axis-I Structured Clinical Interview of the Diagnostic and Statistical Manual, version IV (SCID) and was administered to participants who answered “yes” to either of two screening questions (N=1,626, see Supplementary Materials). Control participants were defined as those individuals who answered “no” to the two screening questions (see Supplementary Materials) or did not fulfil criteria for a diagnosis of current or previous MDD following the SCID interview (N=7,876). Individuals fulfilling criteria for bipolar disorder or those who self-reported either bipolar disorder or schizophrenia (N=11) were excluded.

Educational attainment was measured by asking participants: “What is the highest educational qualification you have obtained?” with nine available answers, detailed in the Supplementary Materials. BMI was computed using height (cm) and weight (kg) as measured by clinical staff at baseline recruitment. Participants reported their smoking status (never, former, current) as well as the number of units of alcohol consumed during the past week. Finally, concentrations of HDL and total cholesterol in blood were measured at baseline by mmol/L.

*ALSPAC*

MDD was measured using the Edinburgh Postnatal Depression Scale (EPDS) (23). Briefly, participants were asked to mark the response closest to how they have been feeling in the past 7 days on a 10-item scale, where the total score is 30 and a score above 13 indicates MDD (23). We transformed the scores into a binary variable, where MDD cases were those who scored above 13 (N=67) and controls were those with a total score of ≤13 (N=498).

BMI was computed using height (cm) and weight (kg) as measured by clinical staff at baseline recruitment. Participants reported whether they currently smoke as well as alcohol consumption frequency (see Supplementary Materials). Concentrations of HDL and total cholesterol were measured by mmol/L. Educational attainment was recorded by asking participants: “What is the highest educational qualification you have obtained?” with six available answers, detailed in the Supplementary Materials.

**DNA methylation**

*GS*

Genome-wide DNAm data profiled from whole blood samples was available for 9,537 individuals in GS using the Illumina Human-MethylationEPIC BeadChip (24). The DNAm data was initially released in two sets (set 1_N_=5,087; set 2_N_=4,450). DNAm data was pre-processed and quality checked for all individuals in the present study, including participant removal due to a number of reasons, including sex mismatch (N_removed_=24), having more than 1% CpG sites with a detection p-value>0.05 (N_removed_=52), showing evidence of dye bias, being an outlier for bisulphite conversion control probes (N_removed_=1), having a median methylated signal intensity more than 3 standard deviations lower than expected (N_removed_=74), and other technical issues (N_removed_=602). A total of 10,495 CpG sites were removed due to low beadcount, poor detection p-value, and sub-optimal binding.

Firstly, R package “minfi” was used to read in the IDAT files, compute M and beta values, and remove probes with large detection p-values, and to compute principal components (PC) of control probes (see Supplementary Tables 2 and 3). Secondly, correction was applied for (1) technical variation, where M values were included as outcome variables in a mixed linear model adjusting for appointment date and Sentrix ID (random effects), jointly with Sentrix position, batch, clinic, year, weekday, and 10 PCs (fixed effects); and (2) biological variation by fitting residuals of (1) as outcome variables in a second mixed linear model adjusting for genetic and common family shared environmental contributions (random effects classed as G: common genetic; K: kinship; F: nuclear family; C: couple; and S: sibling) and sex, age, and estimated cell types proportions (CD8T, CD4T, NK, Bcell, Mono, Gran) (fixed effects) (25).

Cross-reactive (N=42,558) and polymorphic (N=10,971) CpGs, obtained from McCartney et al. (2016) were removed from the final dataset, resulting in 674,246 CpGs across the 22 autosomes (26).

*ALSPAC*

The Illumina Infinium HumanMethylation450 Beadchip (27) was used for measuring genome-wide DNA methylation data from blood sample for all samples. The R package “*mefill*” was used for pre-processing and normalisation (28). Probes were removed based on background detection (p>0.05) and if they reach beyond the 3 times inter-quantile range from 25% and 75%. R function “*beta2m*” from the “*lumi*” package (29) was used for M-value transformation. Cross-reactive and polymorphic CpGs (N=34,881), identified by Chen et al. (2013) were removed, resulting in 447,975 CpGs across the 22 autosomes remaining for analysis (30).

**Statistical methods**

All analyses were conducted using R (version 3.6.3) in a Linux environment. The R code for the current analyses is available in the Supplementary RMarkdown File.

*MDD-relevant factor selection*

To identify risk factors for MDD for building MSs, we first ran logistic regression models to identify nominal associations between environmental, lifestyle, and biochemical variables available in GS, included as predictor variables in separate models, and MDD, included as an outcome variable. The complete list of investigated variables is included in the Supplementary Table 4. Age and sex were included as covariates in all models.

Following this, we conducted a literature search to identify previous MWASs where DNAm signatures of significantly associated factors were uncovered (see Supplementary Materials). To meet inclusion criteria, studies needed to use the 450K or EPIC array in peripheral blood in an adult population; provide access to methylome-wide findings where full summary statistics were not available; and include smoking status as a covariate where relevant. GS was not included in any of the MWASs. ALSPAC was included in the BMI MWAS as a replication cohort (10), however, we used weights from the discovery cohort to calculate the BMI MS. We identified and calculated MSs for six factors: total cholesterol, HDL cholesterol, educational attainment, smoking status, alcohol consumption, and BMI (9–13).

*MS calculation*

All previous MWASs were conducted using the Illumina 450K array. The overlap between CpGs identified in previous MWASs and CpGs available in GS and ALSPAC is presented in Supplementary Table 5. For each trait, MSs were calculated for all individuals in GS and ALSPAC with available DNAm data (N=9,502 and N=565, respectively) by taking the sum of the product of methylome-wide significant CpGs and their estimated weights in each MWAS (9–13). Where full summary statistics were available (educational attainment, smoking status, alcohol consumption), we also calculated MS at 4 further p-value thresholds: 0.01, 0.05, 0.1, and 0.5 (11–13).

In GS, we calculated a MDD MS to compare to each complex trait MS. As there are no previous well-powered MWAS of MDD to date, we split the GS sample into a training (N=5,078) and testing (N=4,432) sample by set, as detailed above. We then applied least absolute shrinkage and selection operator (LASSO) penalised regression on 450K array CpG sites measured in the individuals in the training sample. Briefly, depression status was regressed on age, sex and 10 genetic principal components, as in previous studies (31), and extracted residuals from this model were input as the dependent variable in the regression model. Tenfold cross-validation was applied, and the mixing parameter was set to 1 for our LASSO penalty.

*Association of MS with corresponding traits*

In both cohorts, the associations between each MS and their corresponding traits were assessed using logistic, linear, and ordinal logistic regression (depending on each trait, included as an outcome variable). Each MS was included as a predictor variable in separate models. Technical and biological variables (GS: age and sex; ALSPAC: age, 20 methylation PCs, and estimated proportions for five white blood cell types (CD4T, CD8T, natural killer cells, B-cells, Granulocytes, estimated using the Houseman method (32))) were included as covariates in each model. In GS, methylation PCs and cell type estimations were regressed out during pre-processing of the DNAm data and were therefore not included as covariates in downstream analyses. In ALSPAC, we calculated methylation PCs by first residualizing DNAm data on age, sex, and array, and then applying principal component analysis (PCA) on the residualised data. McFadden’s R^2^ was calculated to determine the proportion of variance in each trait explained by each MS.

*Association of MS with MDD*

We then tested whether each MS was associated with MDD in GS (N=9,502) using logistic regression, where MDD was included as an outcome variable, and each MS was included as a predictor variable. Three statistical models were performed for each MS individually, differing in covariates included. The example below is demonstrated using BMI MS, and these models were repeated for all other MS:

***Model 1****: MDD ~ age + sex + BMI MS*, where the association between each MS and MDD without confounding variables was assessed.

***Model 2****: MDD ~ age + sex + BMI + BMI MS*, where, for each MS, its corresponding phenotype in GS was included to estimate how much variance each MS explains in MDD when adjusting for its corresponding phenotypic measure.

***Model 3****: MDD ~ age + sex + BMI + smoking status + pack years + alcohol consumption + BMI MS*, where further lifestyle factors, known to associate with both MDD and DNAm, were included to observe the proportion of variance explained by the MS when adjusting for these factors.

The same models were run in ALSPAC (N=565), with differing technical and biological covariates: age, 20 methylation PCs, and estimated proportions for five white blood cell types (CD4T, CD8T, natural killer cells, B-cells, granulocytes). BMI, alcohol consumption, and smoking status were included in model 3 as lifestyle factors in ALSPAC.

In a subset of GS (N=4,432), which was created by splitting the sample into a training and testing sample, we further investigated whether an MDD MS would explain more variance in MDD than complex trait MS. The area under the curve was calculated for each MS and a ROC curve showing the ability of each score to discriminate between MDD cases and controls is shown in Figure 3 below.

Finally, we conducted sensitivity analyses by (1) stratifying the GS sample by sex and running models 2 and 3 in a women-only sample (N=5,615, MDD cases=1,163), as the ALSPAC sample consisted of women only; and (2) including smoking status as a covariate in model 1 described above, to observe whether this attenuates the relationship between complex trait MS and MDD due to the widespread effect of smoking on the methylome (33).

*Colocalization analysis*

We hypothesised that some CpG sites included in the complex trait MS will have shared variants with MDD-associated SNPs. We used Howard et al.’s (2019) MDD GWAS for MDD-associated SNPs and GoDMC summary statistics (<http://www.godmc.org.uk/>) for mQTL analysis (2,34). We used the package “*gwasglue”* (<https://mrcieu.github.io/gwasglue/>) to extract SNPs that were +/- 1Mb of each of the 102 genome-wide significant SNPs identified in Howard et al. (2019) and then extracted the same SNPs within those regions from the GoDMC mQTL analysis. We used the “*coloc.abf*” function with default parameters in the “*coloc*” package in R to perform colocalization analysis for each SNP association (35). The method tests for five mutually exclusive scenarios in a genetic region: H_0_: there exist no causal variants for either trait; H_1_: there exists a causal variant for trait one only; H_2_: there exists a causal variant for trait two only; H_3_: there exist two distinct causal variants, one for each trait; and H_4_: there exists a single causal variant common to both traits.

For regions of interest with a posterior probability of >0.5, we performed a manual look-up to identify whether any of the loci in these regions colocalize with genetic variation influencing CpG sites that comprise MS for the 6 complex traits investigated here, including smoking, alcohol consumption, educational attainment, BMI, HDL and total cholesterol.

**Role of funding sources**

Our funding sources were not involved in the study preparation/design, analysis/interpretation of data, or in the writing and submission of this report.

**Results**

*Demographic characteristics*

In GS, there were N=9,502 individuals included in the final analyses (Table 1). In ALSPAC, there were N=565 individuals included in the final analyses (Table 2). Significant differences between cases and controls are indicated in Tables 1 and 2. For model 3, the sample size decreased due to exclusion of individuals who had incomplete lifestyle, disorder, and biochemical data (GS sample for final model=7,890; ALSPAC sample for final model=404). Demographic characteristics for the sub-sample used to test the MDD MS in GS (N=4,432) are included in Supplementary Table 6.

| **Demographic characteristic** | **MDD diagnosis (N=1,626)** | **No MDD Diagnosis (N=7,876)** | **Significance testing** |
| --- | --- | --- | --- |
| *Age (mean, SD) | 48.23 (12.05) | 50.16 (13.83) | t(2614)=5.7, p=1.35x10^-8^ |
| *Sex (%) | F=1,163 (72%) | F=4,452 (57%) | Χ^2^(1)=125.43, p=4.096x10^-29^ |
| *BMI (mean, SD) | 27.41 (5.7) | 26.78 (4.89) | t(2141)= -4.05, p=5.23x10^-5^ |
| Alcohol units (mean, SD) | 10.41 (12.51) | 10.64 (12.09) | t(2009)=0.64, p=0.522 |
| *Smoking status (%) |  |  | Χ^2^(3)=85.76, p=1.78x10^-18^ |
| Current smoker | 395 (24%) | 1,215 (16%) |  |
| Former smokers (quit < 1 year ago) | 47 (3%) | 227 (3%) |  |
| Former smokers (quit > 1 year ago) | 454 (28%) | 2,155 (27%) |  |
| Never smoked tobacco | 696 (43%) | 4,105 (52%) |  |
| Missing | 34 (2%) | 174 (2%) |  |
| *Pack years (mean, SD) | 9.11 (14.18) | 7.66 (14.05) | t(2270)=-3.58, p=3.49x10^-4^ |
| *Educational attainment |  |  | Χ^2^(8)=16.29, p=0.038 |
| No qualification | 134 (8%) | 634 (8%) |  |
| Other | 51 (3%) | 191 (3%) |  |
| School leavers’ certificate | 47 (3%) | 380 (5%) |  |
| CSE/equivalent | 4 (0.25%) | 31 (0.5%) |  |
| Standard grade/O-level/GCSE/equivalent | 192 (11.75%) | 968 (12%) |  |
| Higher grade/A-level/AS-level/equivalent | 150 (9%) | 729 (9%) |  |
| NVQ/HND/HNC/equivalent | 145 (9%) | 646 (8%) |  |
| Other professional/technical qualification | 334 (21%) | 1,561 (19.5%) |  |
| College/University degree | 461 (28%) | 2,190 (28%) |  |
| Missing | 108 (7%) | 546 (7%) |  |
| HDL cholesterol (mean, SD) | 1.48 (0.42) | 1.48 (0.41) | t(2327)=0.14, p=0.891 |
| Total cholesterol (mean, SD) | 5.21 (1.07) | 5.16 (1.06) | t(2331)=-1.62, p=0.105 |

**Table 1.** Demographic characteristics for individuals with an MDD diagnosis and controls in GS (N=9,502); CSE=certificate of secondary education; GCSE=general certificate of secondary education; NVQ=national vocational qualification; HND=higher national diploma; HNC=higher national certificate. *= significant differences between MDD cases and controls.

| **Demographic characteristic** | **MDD diagnosis (N=67)** | **No MDD Diagnosis (N=498)** | **Significance testing** |
| --- | --- | --- | --- |
| Age (mean, SD) | 48.57 (4.5) | 47.95 (4) | t(80.94)=-1.18, p=0.238 |
| BMI (mean, SD) | 25.05 (3.67) | 24.55 (3.33) | t(79.71)=1.30, p=0.2 |
| Smoking status (%) |  |  | Χ^2^(2)=3.49, p=0.174 |
| Current smoker  Never smoked tobacco  Missing | 9 (13%)  58 (87%)  0 (0%) | 35 (7%)  462 (92.8%)  1 (0.2%) |  |
| Alcohol consumption (%) |  |  | Χ^2^(5)=3.32, p=0.651 |
| Never drank | 5 (7%) | 39 (8%) |  |
| Monthly or less | 13 (20%) | 67 (13%) |  |
| 2-4 times/month | 13 (20%) | 92 (18.8%) |  |
| 2-3 times/week | 19 (28%) | 188 (38%) |  |
| 5-4 or more times/week | 17 (25%) | 111 (22%) |  |
| Missing | 0 (0%) | 1 (0.2%) |  |
| *Educational attainment |  |  | Χ^2^(4)=10.37, p=0.035 |
| No qualification  CSE  Vocational  O-level  A-level  Degree | 0 (0%)  8 (12%)  7 (10%)  19 (28%)  23 (34%)  10 (16%) | 0 (0%)  26 (5%)  25 (4.5%)  162 (32.5%)  161 (32%)  124 (26%) |  |
| HDL cholesterol (mean, SD) | 1.45 (0.49) | 1.50 (0.36) | t(85.87)=0.04, p=0.968 |
| Total cholesterol (mean, SD) | 4.87 (0.84) | 4.91 (0.85) | t(104.74)= -0.71, p=0.484 |

**Table 2.** Demographic characteristics for individuals with a MDD diagnosis and controls in ALSPAC (N=565, female only); CSE=certificate of secondary education. *= significant differences between MDD cases and controls.

*MDD-relevant factor selection*

Prior to identifying well-powered MWASs of potential environmental risk factors, in those individuals with available DNAm data in GS (N=9,502) we ran regression models where age and sex were included as covariates, to measure associations of environmental, lifestyle, and biochemical variables with MDD. All variables investigated, as well as results from regression analyses, are available in Supplementary Table 4. Table 3 below indicates results for those variables that were nominally associated with MDD in GS and were also identified as having an established DNAm signature in previous well-powered MWAS. Educational attainment was not associated with MDD in GS, however it has been widely investigated in relation to DNAm and was therefore included in subsequent analyses here.

| **Trait** | **Beta** | **P-value** | **R^2^ (%)** |
| --- | --- | --- | --- |
| HDL cholesterol | -0.116 | 0.0001 | 0.9% |
| Total cholesterol | 0.069 | 0.016 | 0.6% |
| Smoking status | 0.567 | 1.13x10^-16^ | 3.2% |
| Alcohol (units) | 0.103 | 0.0007 | 10.8% |
| BMI | 0.149 | 1x10^-8^ | 0.9% |
| Educational attainment | -0.003 | 0.766 | 0.6% |

**Table 3.** Associations between environmental and biochemical factors and MDD in GS (N=9,502) in logistic regression models. All variables are significantly associated with MDD apart from educational attainment.

*MS and corresponding traits*

Each MS, which was included as a predictor, was first investigated in relation to its corresponding phenotype, as outcome, in regression models, along with technical and biological covariates (GS: age and sex; ALSPAC: age, 20 methylation PCs, and 5 cell types (CD4T, CD8T, natural killer cells, B-cells, granulocytes)). All MSs were associated with their phenotypic counterparts in both GS and ALSPAC. R^2^ for all analyses are presented in Supplementary Figures 1 and 2. Tables 4 and 5 present results for both cohorts.

*GS*

| **MS** | **Outcome** | **Beta** | **P-value** | **R^2^ (%)** |
| --- | --- | --- | --- | --- |
| **HDL cholesterol (MW)** | HDL cholesterol | 0.189 | < 2x10^-16^ | 3.5% |
| **Total cholesterol (MW)** | Total cholesterol | 0.117 | < 2x10^-16^ | 1.4% |
| **BMI (MW)** | BMI | 0.407 | < 2x10^-16^ | 16.5% |
| **Educational attainment** |  |  |  |  |
| MW | Educational attainment | 0.313 | 2.6x10^-59^ | 1.25% |
| 0.01 |  | 0.278 | 2.04x10^-46^ | 1.07% |
| 0.05 |  | 0.243 | 5.99x10^-36^ | 0.93% |
| 0.1 |  | 0.225 | 3.03x10^-31^ | 0.86% |
| 0.5 |  | 0.203 | 9.13x10^-26^ | 0.78% |
| **Smoking status** |  |  |  |  |
| MW | Smoking status | 1.457 | < 2x10^-16^ | 24.1% |
| 0.01 |  | 1.251 | < 2x10^-16^ | 18.8% |
| 0.05 |  | 1.158 | < 2x10^-16^ | 16.6% |
| 0.1 |  | 1.120 | < 2x10^-16^ | 15.7% |
| 0.5 |  | 1.040 | < 2x10^-16^ | 13.8% |
| **Alcohol units** |  |  |  |  |
| MW | Alcohol units | 0.244 | < 2x10^-16^ | 5.9% |
| 0.01 |  | 0.137 | 1.69x10^-42^ | 1.9% |
| 0.05 |  | 0.114 | 9.82x10^-30^ | 1.3% |
| 0.1 |  | 0.105 | 2.59x10^-25^ | 1.1% |
| 0.5 |  | 0.089 | 9.03x10^-19^ | 0.8% |

**Table 4**. Associations between environmental factors (outcome) and their corresponding MS in GS (N=9,502), where age and sex were included as covariates, in linear, logistic, and ordinal regression models. Where available (educational attainment, smoking status, alcohol units), associations are presented for MS calculated at multiple significance thresholds (p=methylome-wide (MW, Bonferroni-corrected CpGs), <0.01, <0.05, <0.1, <0.5).

*ALSPAC*

| **MS** | **Outcome** | **Beta** | **P-value** | **R^2^ (%)** |
| --- | --- | --- | --- | --- |
| **HDL cholesterol (MW)** | HDL cholesterol | 0.078 | 0.008 | 1.062% |
| **Total cholesterol (MW)** | Total cholesterol | -0.116 | 0.003 | 1.322% |
| **BMI (MW)** | BMI | 1.179 | 1.28x10^-6^ | 4.82% |
| **Educational attainment** |  |  |  |  |
| MW | Educational attainment | 0.236 | 0.004 | 5.26% |
| 0.01 |  | 0.268 | 0.008 | 5.16% |
| 0.05 |  | 0.199 | 0.071 | 4.93% |
| 0.1 |  | 0.248 | 0.031 | 5.01% |
| 0.5 |  | 0.347 | 0.005 | 5.21% |
| **Smoking status** |  |  |  |  |
| MW | Smoking status | -2.533 | 4.30x10^-14^ | 19.08% |
| 0.01 |  | -2.401 | 1.05x10^-12^ | 12.75% |
| 0.05 |  | -2.302 | 2.07x10^-11^ | 10.79% |
| 0.1 |  | -2.224 | 1.31x10^-10^ | 9.85% |
| 0.5 |  | -1.991 | 1.08x10^-8^ | 7.67% |
| **Alcohol units** |  |  |  |  |
| MW | Alcohol units | 0.660 | 1.02x10^-8^ | 3.26% |
| 0.01 |  | 0.593 | 3.76x10^-6^ | 2.73% |
| 0.05 |  | 0.543 | 1.88x10^-5^ | 2.53% |
| 0.1 |  | 0.510 | 4.76x10^-5^ | 2.43% |
| 0.5 |  | 0.415 | 5.42x10^-4^ | 2.20% |

**Table 5**. Associations between environmental factors (outcome) and their corresponding MSs in ALSPAC (N=565), where age, 20 methylation PCs, and 5 cell types were included as covariates, in linear, logistic, and ordinal regression models. Where available (educational attainment, smoking status, alcohol units), associations are presented for MS calculated at multiple significance thresholds (p=methylome-wide (MW, Bonferroni-corrected CpGs), <0.01, <0.05, <0.1, <0.5).

*MS and MDD*

We then examined each MS, as a predictor, with MDD as the outcome, in logistic regression models. Table 6 includes results from the regression model 1 (covariates: GS: age, sex; ALSPAC: age, 20 methylation PCs, and 5 cell types), model 2 (covariates: model 1+corresponding phenotype for each MS for both cohorts) and model 3 (covariates: GS: model 2+4 lifestyle factors; ALSPAC: model 2+3 lifestyle factors). Figures 1 and 2 include R^2^ for models 2 and 3 in GS and ALSPAC, respectively. Supplementary Tables 7 and 8 include R^2^ for all models in GS and ALSPAC, respectively.

As the replication analyses in ALSPAC consisted of women only, we further stratified the GS sample by sex and ran models 2 and 3 in a women-only sample (N=5,615, MDD cases=1,163), with results available in Supplementary Table 9. Briefly, analyses restricted to women in GS showed similar results to the sex-adjusted analyses in GS, where MS were associated with MDD after adjustment for their phenotypic counterparts, but not when including further lifestyle factors.

In addition, due to the known effects of smoking on the methylome (33), we included smoking status as a covariate in model 1 for all non-smoking traits to identify whether this attenuates the relationship between MDD and complex trait MS without adjusting for other covariates. Results are available in Supplementary Table 10. In both GS and ALSPAC, the effect for all complex trait MS was attenuated by the inclusion of smoking. In GS, all complex trait MS remained significant in their association with MDD, except for educational attainment. In ALSPAC, results remained non-significant as below.

|  | **GS** | | | | | |
| --- | --- | --- | --- | --- | --- | --- |
|  | **Model 1** | | **Model 2** | | **Model 3** | |
| **MS** | **Beta** | **P-value** | **Beta** | **P-value** | **Beta** | **P-value** |
| **HDL cholesterol (MW)** | **-0.113** | **3.81x10^-5^** | **-0.097** | **0.0006** | -0.062 | 0.05 |
| **Total cholesterol (MW)** | **-0.077** | **0.005** | **-0.086** | **0.002** | -0.043 | 0.167 |
| **BMI (MW)** | **0.138** | **3.83x10^-7^** | **0.092** | **0.002** | 0.051 | 0.128 |
| **Educational attainment** |  |  |  |  |  |  |
| MW | **-0.142** | **9.77x10^-8^** | **-0.145** | **3.59 x10^-7^** | **0.094** | **0.038** |
| 0.01 | **-0.148** | **7.25 x10^-8^** | **-0.152** | **2.05 x10^-7^** | -0.044 | 0.198 |
| 0.05 | **-0.125** | **6.30 x10^-6^** | **-0.129** | **1.09 x10^-5^** | -0.042 | 0.203 |
| 0.1 | **-0.120** | **1.65 x10^-5^** | **-0.124** | **2.19 x10^-5^** | -0.046 | 0.153 |
| 0.5 | **-0.109** | **7.37 x10^-5^** | **-0.117** | **6.45 x10^-5^** | -0.052 | 0.109 |
| **Smoking status** |  |  |  |  |  |  |
| MW | **0.160** | **2.89 x10^-9^** | 0.053 | 0.095 | 0.033 | 0.334 |
| 0.01 | **0.159** | **4.01 x10^-9^** | **0.070** | **0.022** | 0.050 | 0.128 |
| 0.05 | **0.157** | **7.58 x10^-9^** | **0.074** | **0.014** | 0.054 | 0.099 |
| 0.1 | **0.155** | **1.14 x10^-8^** | **0.075** | **0.013** | 0.054 | 0.094 |
| 0.5 | **0.148** | **4.83 x10^-8^** | **0.072** | **0.015** | 0.052 | 0.104 |
| **Alcohol units** |  |  |  |  |  |  |
| MW | **0.061** | **0.03** | **0.059** | **0.044** | 0.018 | 0.561 |
| 0.01 | **-0.061** | **0.03** | **-0.066** | **0.031** | **-0.069** | **0.026** |
| 0.05 | **-0.083** | **0.004** | **-0.085** | **0.005** | **-0.079** | **0.010** |
| 0.1 | **-0.089** | **0.002** | **-0.090** | **0.003** | **-0.082** | **0.008** |
| 0.5 | **-0.097** | **0.0006** | **-0.097** | **0.001** | **-0.083** | **0.007** |
|  | **ALSPAC** | | | | | |
|  | **Model 1** | | **Model 2** | | **Model 3** | |
| **MS** | **Beta** | **P-value** | **Beta** | **P-value** | **Beta** | **P-value** |
| **HDL cholesterol (MW)** | -0.149 | 0.557 | -0.103 | 0.691 | 0.182 | 0.573 |
| **Total cholesterol (MW)** | 0.029 | 0.855 | 0.017 | 0.917 | 0.125 | 0.512 |
| **BMI (MW)** | -0.04 | 0.823 | -0.129 | 0.555 | -0.114 | 0.593 |
| **Educational attainment** |  |  |  |  |  |  |
| MW | -0.23 | 0.064 | -0.174 | 0.171 | -0.214 | 0.324 |
| 0.01 | -0.231 | 0.195 | -0.171 | 0.349 | -0.208 | 0.348 |
| 0.05 | -0.33 | 0.105 | -0.285 | 0.166 | -0.3 | 0.22 |
| 0.1 | -0.359 | 0.092 | -0.308 | 0.154 | -0.314 | 0.218 |
| 0.5 | **-0.483** | **0.03** | -0.41 | 0.068 | -0.474 | 0.079 |
| **Smoking status** |  |  |  |  |  |  |
| MW | 0.287 | 0.104 | 0.214 | 0.301 | 0.435 | 0.065 |
| 0.01 | 0.32 | 0.141 | 0.232 | 0.331 | 0.447 | 0.106 |
| 0.05 | 0.293 | 0.205 | 0.2 | 0.418 | 0.401 | 0.164 |
| 0.1 | 0.277 | 0.239 | 0.186 | 0.456 | 0.373 | 0.201 |
| 0.5 | 0.24 | 0.319 | 0.155 | 0.536 | 0.313 | 0.288 |
| **Alcohol units** |  |  |  |  |  |  |
| MW | 0.283 | 0.156 | 0.282 | 0.162 | **0.494** | **0.042** |
| 0.01 | -0.03 | 0.899 | -0.038 | 0.874 | 0.184 | 0.508 |
| 0.05 | -0.09 | 0.715 | -0.096 | 0.7 | 0.125 | 0.665 |
| 0.1 | -0.096 | 0.698 | -0.102 | 0.685 | 0.108 | 0.712 |
| 0.5 | -0.099 | 0.688 | -0.101 | 0.684 | 0.06 | 0.838 |

**Table 6**. Associations between MDD and MS in GS and ALSPAC across three incremental models differing in covariates included (model 1 covariates: GS (N=9,502): age, sex; ALSPAC (N=565): age, 20 methylation PCs, and 5 cell types; model 2 covariates: model 1+corresponding phenotype for each MS for both cohorts; model 3 covariates: GS (N=7,890): model 2+4 lifestyle factors; ALSPAC (N=404): model 2+3 lifestyle factors), in logistic regression models. Where available (educational attainment, smoking status, alcohol units), associations are presented for MS calculated at multiple significance thresholds (p=methylome-wide (MW, Bonferroni-corrected CpGs), <0.01, <0.05, <0.1, <0.5). Statistically significant results are represented in bold.

*MS and MDD – subset analysis*

To investigate whether an MDD MS would out-perform complex trait MSs in the discrimination between MDD cases and controls, we additionally trained an MDD MS in a subset of individuals with DNAm data in GS (N=5,078, MDD=1,223), where 78 CpGs were selected (Supplementary Table 11). We then calculated an MDD MS in a second subset (N=4,432, MDD=408). See Figure 3 for a Receiver Operating Characteristic (ROC) curve showing the ability of each complex trait MS and MDD MS to discriminate between MDD cases and controls. The two complex trait MS that outperformed MDD MS performance (AUC=0.553) are smoking (AUC=0.569) and educational attainment (AUC=0.585). We applied a DeLong test to identify whether this outperformance is statistically significant. Educational attainment and smoking MS were both non-significant when compared to a MDD MS in predicting MDD: educational attainment D=1.814, p=0.07; smoking D=0.913, p=0.36, indicating that although the AUC for the two complex traits is higher, the difference is not statistically significant.

*Colocalization analysis*

Colocalization analysis indicated that there was no strong evidence (PP_4_>0.8, PP_4_/PP_3_ >5 (36)) for a single SNP being associated with both MDD and DNAm at CpGs encompassing the MS. The posterior probability for one region was supportive of a suggestive co-localized association signal for both MDD and DNAm in that region (PP_4_=0.71) (37). Within this region, the SNP with the highest posterior probability of being a causal SNP (66%) was rs73163796, which colocalized with genetic variation influencing a smoking-associated CpG site, cg15099418 (13). Supplementary Excel File 1 contains results for all 102 regions investigated in colocalization analysis.

**Discussion**

We created MSs for 6 environmental and biochemical risk factors for MDD, namely HDL and total cholesterol, BMI, educational attainment, smoking status, and alcohol consumption, in two cohorts, GS (N=9,502) and ALSPAC (N=565). Methylome-wide scores, and where available, scores at multiple p-value thresholds (educational attainment, smoking status, and alcohol consumption), showed significant associations with their corresponding traits and with MDD after adjustment for their phenotypic counterparts. Most findings attenuated and became non-significant after adjustment for further lifestyle factors. Smoking and education MS marginally outperformed a MDD MS in discriminating between MDD cases and controls in a GS sub-sample (N=4,432). Finally, colocalization analysis showed that genetic variants are shared between a smoking associated CpG site (cg15099418) and MDD.

Each MS was significantly associated with its corresponding phenotype in both cohorts (GS β_range_=0.089-1.457; ALSPAC β_range_=0.078-2.533). All training MWASs consisted of large sample sizes (N_range_=725 (HDL and total cholesterol) – 15,907 (smoking status)), included relevant covariates, and results were consistent where replication cohorts were included (see Supplementary Table 1). All of the training MWAS for phenotypes investigated here were sufficiently predictive of the trait in our independent samples (see Tables 4 and 5). The variance explained by MS was 5% for HDL cholesterol and 12%-27.3% alcohol consumption in Braun et al. (2017) (9) and Liu et al. (2016) (11), respectively. Other studies that applied penalised regression to derive methylation predictors of environmental factors identified similar proportions of variance explained: 12.5% for BMI and alcohol consumption, 60.9% for smoking, 2.5% for educational attainment, 2.7% for total cholesterol, and 15.6% for HDL cholesterol (14). These results are consistent with findings here. In contrast, we previously found that a MDD MS explains 1.75% of the variance in MDD, and attenuates when including lifestyle factors (0.68%) (7). This indicates that, although there is evidence of an association between DNAm and MDD, the relationship is not as strong as with lifestyle factors, which is in line with previous evidence (14).

MSs calculated at different p-value thresholds (methylome-wide, 0.01, 0.05, 0.1, 0.5) indicated that the most predictive threshold for each trait was the most conservative one. CpGs meeting a less stringent p-value threshold in the score captured less phenotypic variance for each corresponding trait (R^2^: smoking=24.1% and 19.08% for methylome-wide threshold compared to 13.8% and 7.67% for p<0.05 threshold in GS and ALSPAC, respectively; education=1.25% and 5.26% for methylome-wide threshold compared to 0.78% and 5.21% for p<0.05 threshold in GS and ALSPAC, respectively; alcohol=5.9% and 3.26% for methylome-wide threshold compared to 0.8% and 2.20% for p<0.05 threshold in GS and ALSPAC, respectively). Consistent with this pattern, previous studies suggest that the optimal p-value threshold strongly depends on the epigenetic architecture of the trait, as well as on the strength of supporting data (38). Lifestyle traits such as smoking, alcohol, and BMI show widespread associations with peripheral blood DNAm (9–13), and indeed MWAS for lifestyle traits investigated here have large sample sizes as well as the largest number of associated CpGs at a methylome-wide threshold. All CpGs significantly associated with educational attainment (12) were also found to be associated with smoking (13), which may explain the similar association pattern to lifestyle factors.

In GS, each MS was significantly associated with MDD before corresponding trait and lifestyle factor adjustment, although this was only replicated for methylome-wide educational attainment in ALSPAC. When including smoking status in the covariate-free MS-MDD associations, the effects for all complex trait MSs were attenuated but, with the exception of educational attainment, remained significant (Supplementary Table 10). Although the CpGs (N=11) associated with educational attainment, were adjusted for smoking status in the original MWAS (12), all 11 were also smoking-associated CpGs, indicating that the two traits share an epigenetic signature and that self-reported smoking status may not be sufficient to correct for smoking signals (33). In GS, the epigenetic signature of each trait explained additional variance to its phenotypic counterpart in MDD, although effect sizes were small across all traits (methylome-wide_βrange_=0.053-0.145). For traits where MSs were available at multiple p-value thresholds, the variance explained increased for MSs that included CpGs at a larger p-value threshold for smoking (R^2^=0.03% methylome-wide MS to 0.07% p<0.05) and alcohol consumption (R^2^=0.05% methylome-wide MS to 0.1% p<0.05) and decreased for educational attainment (R^2^=0.28% methylome-wide MS to 0.20% p<0.05). This suggests that for lifestyle traits with widespread effects on the methylome, including a larger number of associated CpG sites increased prediction accuracy for MDD, although the effect size did not differ significantly (smoking_β methylome-wide MS_=0.053 to smoking_β p<0.05_=0.072; alcohol consumption_β methylome-wide MS_=0.059 to alcohol consumption_β p<0.05_=-0.097; educational attainment_methylome-wide MS_=-0.145 to educational attainment_β p<0.05_=-0.117).

After adjusting for lifestyle factors that are known to associate with MDD and DNAm (smoking, pack years, BMI, and alcohol consumption), methylome-wide educational attainment and alcohol consumption at 4 p-value thresholds (p<0.01-0.5) remained significantly associated with MDD, suggesting that certain lifestyle factors may attenuate the relationship between epigenetic signatures of specific traits and MDD. This is surprising given that smoking is known to have much larger effects on the methylome than alcohol consumption (13). However, smoking was included as a covariate in all previous MWASs used for the MSs calculated here, while the smoking MWAS (13) did not adjust for any lifestyle factors, suggesting that the smoking MS captures other environment-related CpGs whose effect is attenuated by phenotypic measures of lifestyle factors. This pattern of results is consistent with previous studies where disease-relevant phenotypes attenuate associations between MS for complex traits and disease. For instance, Yu et al. (2020) investigated associations between smoking MS and lung cancer before and after adjustment for phenotypic smoking status and pack years. They found that the phenotypes attenuated the association between smoking MS and lung cancer, with odds ratio decreasing across different risk score quartiles (15).

Here, we showed that MS for complex traits enhance MDD risk prediction when added to phenotypic measures of these traits. DNA methylation may represent an archive of exposure to environmental factors that are relevant to MDD and may contribute to disease vulnerability. However, lifestyle factors may play an important role in this relationship. Here, they were shown to attenuate the association between MDD and complex trait MS, indicating that they may interact with widespread DNAm in their association with MDD. This is not surprising, as we have previously found that a MDD MS was significantly associated with smoking and alcohol consumption (7). Although in our previous study the MDD MS enhanced MDD risk prediction when modelled alongside lifestyle factors, here complex trait MS effects are attenuated in the same scenario. This may show that phenotypic measures of lifestyle factors play a greater role in MDD development than methylation marks for complex traits. However, effect sizes here were small, and further studies will be needed to determine the extent of the role DNA methylation plays in MDD.

While the association between each MS and their corresponding phenotype was replicated in ALSPAC, analyses investigating MDD were not. When the MS was modelled together with its phenotypic counterpart, effects were in the same direction with GS across all traits with the exception of BMI, which was positive in GS but negative in ALSPAC. The opposite pattern was shown in terms of variance explained for MS calculated at multiple thresholds, where explained variance in MDD decreased with a less stringent threshold for smoking (R^2^=0.06% methylome-wide MS to 0% p<0.05) and alcohol consumption (R^2^=0.32% methylome-wide MS to 0% p<0.05) and increased for educational attainment (R^2^=0.14% methylome-wide MS to 0.97% p<0.05). The variance explained in MDD did not exceed 1% for any of the traits in GS. Larger cohorts may, therefore, be required to elucidate the link between MDD and epigenetic signatures of lifestyle factors.

The MWASs used to create MS in the current study uncovered CpGs localised to a number of genes that may be of relevance to MDD. The MWAS of educational attainment identified genes implicated in neuronal, immune, and developmental processes (12); alcohol consumption-associated CpGs were localised to genes involved in cellular response to stress and chemicals, and immune functions (11); BMI-associated CpGs were linked to genes that played a role in lipid metabolism, inflammation, metabolic, cardiovascular, respiratory, and neoplastic disease (10); smoking-related methylation marks were localised to genes implicated in smoking-related diseases (osteoporosis, colorectal cancers, chronic obstructive pulmonary disease, pulmonary function, cardiovascular disease, rheumatoid arthritis) (13); finally, HDL and total cholesterol-associated CpGs were annotated to genes implicated in cholesterol metabolism (9). Most processes identified in these MWAS have also been previously associated with MDD and antidepressant use, specifically immune and neuronal processes (2,39). It is therefore possible that some associations between MDD and complex trait MS in this study may arise as a result of the processes in which the genes above participate, although further studies are needed to confirm this.

Differences in results between the two cohorts may be attributable to sample size (N_GS_=9,502; N_ALSPAC_=565) and phenotypic differences. Firstly, the ALSPAC sample consists of women only. However, analyses restricted to women in GS (N=5,615) showed similar results to the sex-adjusted analyses in GS (see Supplementary Table 9), indicating that the lack of replication may be due to factors such as the much smaller sample size in ALSPAC (N=565) rather than sex. Further, although the replication sample was matched in age (GS_mean age_=49.82, ALSPAC_mean age_=47.96), there were differences in lifestyle factors between the two cohorts. For instance, 18% of participants in GS smoked at the time of blood draw, as opposed to 8% in ALSPAC; 28% and 24% of individuals held a university degree in GS and ALSPAC, respectively. In addition to this, all participants in ALSPAC had some form of education qualification, whereas 8% of GS participants held no qualifications. Finally, BMI was lower in ALSPAC (mean=24.99) as compared to GS (mean=26.89).

Further, similarly to GS, all MSs in ALSPAC explained a significant proportion of variance in their corresponding phenotypic traits, with non-replicating analyses occurring only when MDD was investigated. MDD was assessed differently in the two cohorts: in GS, this was measured using SCID, while in ALSPAC, MDD status was determined by classifying participants with a score of >13 on the EPDS as cases. Previous studies have shown that EPDS approximated SCID-based prevalence overall, although considerable heterogeneity between cohorts may play a role in this approximation (40).

A MDD MS tested in a GS sub-sample (N=4,432) was outperformed by smoking and education MSs in predicting MDD, although predictive values were low for all MS (MDD_AUC_=0.553, smoking_AUC_=0.569, education_AUC_=0.585). DNAm is highly predictive of smoking (13), and there is a strong overlap of smoking-associated CpGs in the educational attainment MWAS used to calculate the MS (12). The two predictors are also highly correlated (*r*=-0.720). Results therefore suggest that epigenetic signatures of lifestyle traits showing more widespread associations with DNAm are marginally more predictive of MDD than an MDD-specific predictor, although current results are limited by lack of large MWAS of MDD.

There are several key strengths to this study. Firstly, GS is one of the largest population-based cohorts containing DNAm and a broad range of lifestyle, disorder, and environmental variables. Secondly, this study provides insight into associations between MSs for lifestyle and biochemical factors in relation to MDD across multiple p-value thresholds. Finally, we used a second large, population-based study as a replication cohort, which similarly contains a range of lifestyle and environmental variables, in addition to DNAm.

Despite these strengths, a number of potential limitations to the current study also need to be considered. Firstly, although GS uses the EPIC array, capturing DNAm at approximately 850K sites, previous MWAS are limited by use of the 450K array, which measures methylation at 450K CpGs. Using a larger array may improve the predictive accuracy for environmental traits, which in turn may lead to more precise associations in relation to MDD. Secondly, DNAm was collected from blood samples in both cohorts and in all previous MWASs, which may not be the most relevant tissue for MDD. However, previous studies have shown robust associations between peripheral blood-based methylation predictors and MDD (6,41,42). Participants in GS and ALSPAC are predominantly of European ancestry, and the generalisability to diverse ancestries is unknown. Finally, as mentioned above, ALSPAC contained only women who smoked less and had a lower BMI than GS participants; GS participants reported a higher level of educational attainment than ALSPAC women. Although stratifying the GS cohort by women only indicated that results are not due to sex differences, results here may be due to these phenotypic differences, and future studies should select replication cohorts that are analogous to the training cohort.

In the current study we showed that epigenetic signatures of lifestyle and biochemical factors are associated with MDD after adjustment for their phenotypic counterparts, but not when including a broader number of lifestyle factors. Results were not replicated in a second cohort, which may be due to phenotypic differences compared to the main cohort as well as the much smaller sample size. Lifestyle variables are significant in terms of DNAm-related risk to MDD, and efforts should be made in future to disentangle the relationship between these lifestyle factors, DNAm, and MDD. Our study demonstrates the value and necessity of large DNAm datasets for discovery and replication within and between cohorts.

**Contributors**

MCB, HCW, and AMM were responsible for the project conceptualisation, methodology and validation. MCB, ASFK, MJA, and AC carried out the data curation. CA, RMW, SWM, and KLE were responsible for DNA methylation data quality check and pre-processing in Generation Scotland and ALSPAC. CL, JvD, and MG were responsible for providing summary statistics for the current study. JLM was responsible for providing results in the form of summary statistics (GoDMC). CR and DJP are project directors for ALSPAC and Generation Scotland, respectively. MCB, HCW, and AMM were responsible for the decision to submit. MCB was responsible for formal analysis and writing the original draft and visualisation. XS additionally verified the underlying data and analysis. MCB, CA, ASFK, XS, MJA, DMH, RMW, SWM, JLM, CL, JvD, MG, CR, DJP, AC, KLE, HCW, and AMM reviewed versions of the manuscript. MCB, HCW, and AMM were responsible for manuscript editing and review. AMM and HCW were responsible for supervision, project administration, resources, and funding acquisition. MCB, CA, ASFK, XS, MJA, DMH, RMW, SWM, JLM, CL, JvD, MG, CR, DJP, AC, KLE, HCW, and AMM were not precluded from accessing data in the study and they accept responsibility to submit for publication. All authors read and approved the final version of the manuscript.

**Declaration of interests**

MCB has received financial support from Edinburgh Neuroscience Researcher’s Fund, Wellcome Trust Institutional Translational Partnership Award Innovation Competition, and Research Adaptation Fund to attend courses and conferences in the past. RMW has received financial support from Alzheimer’s Research UK (ARUK) to attend the ARUK annual conference (2021 and 2022). JLM is supported by the UK Medical Research Council Integrative Epidemiology Unit at the University of Bristol. AC is a University of Edinburgh Medical Research Ethics Committee member. JvdD was supported by NWO Large Scale infrastructures, X-Omics (184.034.019). Remaining authors report no conflicts of interest.

**Acknowledgments**

This research was funded in whole, or in part, by the Wellcome Trust [216767/Z/19/Z]. For the purpose of open access, the author has applied a CC BY public copyright licence to any Author Accepted Manuscript version arising from this submission. Generation Scotland is currently supported by the Wellcome Trust [216767/Z/19/Z] and by the Wellcome Trust Investigator Award in Science 01/06/2021 to 31/05/26 ‘Exploiting genomic approaches to identify the environmental basis of depression’. (Reference: 220857/Z/20/Z) to McIntosh AM (PI). The DNA methylation profiling and data preparation was supported by Wellcome Investigator Award 220857/Z/20/Z and Grant 104036/Z/14/Z (PI for both grants: McIntosh AM) and through funding from NARSAD (Ref: 27404; PI: Dr DM Howard and Ref: 21956; PI Dr Kathryn Evans) and the Royal College of Physicians of Edinburgh (Sim Fellowship; PI: Dr HC Whalley). Genotyping of the GS:SFHS samples was funded by the MRC and Wellcome Trust [104036/Z/14/Z]. Generation Scotland also receives support from the Chief Scientist Office of the Scottish Government Health Directorates [CZD/16/6] and the Scottish Funding Council [HR03006]. Dr DM Howard is supported by a Sir Henry Wellcome Postdoctoral Fellowship (Reference 213674/Z/18/Z). Dr M Barbu is supported by a Guarantors of Brain Non-clinical Post-Doctoral Fellowship.

The UK Medical Research Council and Wellcome (Grant Ref: 217065/Z/19/Z) and the University of Bristol provide core support for ALSPAC. A comprehensive list of grants funding is available on the ALSPAC website (<http://www.bristol.ac.uk/alspac/external/documents/grant-acknowledgements.pdf>).

We are extremely grateful to all the families who took part in this study, the midwives for their help in recruiting them, and the whole ALSPAC team, which includes interviewers, computer and laboratory technicians, clerical workers, research scientists, volunteers, managers, receptionists and nurses. Part of this data was collected using REDCap, see the REDCap website for details <https://projectredcap.org/resources/citations/>).

**Data sharing statement**

Data used in the preparation of this article were obtained from the Generation Scotland (GS) cohort (<https://www.ed.ac.uk/generation-scotland>) and the Avon Longitudinal Study of Parents and Children (ALSPAC) cohort (<http://www.bristol.ac.uk/alspac/>).

Qualified researchers can request access to GS data through a research proposal, accessed at the following link: <https://www.ed.ac.uk/generation-scotland/for-researchers/access> and to ALSPAC, accessed at the following link: <http://www.bristol.ac.uk/alspac/researchers/access/>.

Scripts for the analyses in this project can be found in the Supplementary RMarkdown file.

**References**

1. Mcintosh AM, Sullivan PF, Lewis CM. Review Uncovering the Genetic Architecture of Major Depression. Neuron. 2019;102(1):91–103.

2. Howard DM, Adams MJ, Clarke TK, Hafferty JD, Gibson J, Shirali M, et al. Genome-wide meta-analysis of depression identifies 102 independent variants and highlights the importance of the prefrontal brain regions. Nat Neurosci. 2019 Mar;22(3):343–52.

3. Sarris J, Thomson R, Hargraves F, Eaton M, de Manincor M, Veronese N, et al. Multiple lifestyle factors and depressed mood: a cross-sectional and longitudinal analysis of the UK Biobank (N = 84,860). BMC Med. 2020 Dec;18(1):354.

4. Choi KW, Stein MB, Nishimi KM, Ge T, Coleman JRI, Chen CY, et al. An exposure-wide and mendelian randomization approach to identifying modifiable factors for the prevention of depression. Am J Psychiatry. 2020 Oct;177(10):944–54.

5. Jovanova OS, Nedeljkovic I, Spieler D, Walker RM, Liu C, Luciano M, et al. DNA methylation signatures of depressive symptoms in middle-aged and elderly persons: Meta-analysis of multiethnic epigenome-wide studies. JAMA Psychiatry. 2018 Sep;75(9):949–59.

6. Starnawska A, Tan Q, Soerensen M, McGue M, Mors O, Børglum AD, et al. Epigenome-wide association study of depression symptomatology in elderly monozygotic twins. Transl Psychiatry. 2019 Dec;9(1):1–14.

7. Barbu MC, Shen X, Walker RM, Howard DM, Evans KL, Whalley HC, et al. Epigenetic prediction of major depressive disorder. Mol Psychiatry. 2020;

8. Clark SL, Hattab MW, Chan RF, Shabalin AA, Han LKM, Zhao M, et al. A methylation study of long-term depression risk. Mol Psychiatry. 2020 Jun;25(6):1334–43.

9. Braun KVE, Dhana K, de Vries PS, Voortman T, van Meurs JBJ, Uitterlinden AG, et al. Epigenome-wide association study (EWAS) on lipids: the Rotterdam Study. Clin Epigenetics. 2017 Feb;9(1):15.

10. Wahl S, Drong A, Lehne B, Loh M, Scott WR, Kunze S, et al. Epigenome-wide association study of body mass index, and the adverse outcomes of adiposity. Nature. 2017 Jan;541(7635):81–6.

11. Liu C, Marioni RE, Hedman AK, Pfeiffer L, Tsai PC, Reynolds LM, et al. A DNA methylation biomarker of alcohol consumption. Mol Psychiatry. 2018 Feb;23(2):422–33.

12. van Dongen J, Bonder MJ, Dekkers KF, Nivard MG, van Iterson M, Willemsen G, et al. DNA methylation signatures of educational attainment. npj Sci Learn. 2018 Dec;3(1):7.

13. Joehanes R, Just AC, Marioni RE, Pilling LC, Reynolds LM, Mandaviya PR, et al. Epigenetic Signatures of Cigarette Smoking. Circ Cardiovasc Genet. 2016;436–47.

14. McCartney DL, Hillary RF, Stevenson AJ, Ritchie SJ, Walker RM, Zhang Q, et al. Epigenetic prediction of complex traits and death. Genome Biol. 2018 Sep;19(1):136.

15. Yu H, Yu H, Raut JR, Raut JR, Schöttker B, Schöttker B, et al. Individual and joint contributions of genetic and methylation risk scores for enhancing lung cancer risk stratification: data from a population-based cohort in Germany. Clin Epigenetics. 2020 Jun;12(1):1–11.

16. Battram T, Yousefi P, Crawford G, Prince C, Babei MS, Sharp G, et al. The EWAS Catalog: a database of epigenome-wide association studies.

17. Smith BH, Campbell H, Blackwood D, Connell J, Connor M, Deary IJ, et al. Generation Scotland: The Scottish Family Health Study; a new resource for researching genes and heritability. BMC Med Genet. 2006 Oct;7(1):74.

18. Smith BH, Campbell A, Linksted P, Fitzpatrick B, Jackson C, Kerr SM, et al. Cohort Profile : Generation Scotland : Scottish Family Health Study ( GS : SFHS ). The study , its participants and their potential for genetic research on health and illness. Int J Epidemiol. 2013;(July 2012):689–700.

19. Fraser A, Macdonald-wallis C, Tilling K, Boyd A, Golding J, Davey smith G, et al. Cohort profile: The avon longitudinal study of parents and children: ALSPAC mothers cohort. Int J Epidemiol. 2013 Feb;42(1):97–110.

20. Boyd A, Golding J, Macleod J, Lawlor DA, Fraser A, Henderson J, et al. Cohort Profile: The ‘Children of the 90s’—the index offspring of the Avon Longitudinal Study of Parents and Children. Int J Epidemiol. 2013 Feb;42(1):111–27.

21. PA H, R T, R T, J P, N G, JG C. Research electronic data capture (REDCap)--a metadata-driven methodology and workflow process for providing translational research informatics support. J Biomed Inform. 2009 Apr;42(2):377–81.

22. Relton CL, Gaunt T, McArdle W, Ho K, Duggirala A, Shihab H, et al. Data Resource Profile: Accessible Resource for Integrated Epigenomic Studies (ARIES). Int J Epidemiol. 2015 Aug;44(4):1181–90.

23. Cox JL, Holden JM, Sagovsky R. Detection of Postnatal Depression: Development of the 10-item Edinburgh Postnatal Depression scale. Br J Psychiatry. 1987;150(JUNE):782–6.

24. Hansen K. IlluminaHumanMethylationEPICanno.ilm10b2.hg19: Annotation for Illumina’s EPIC methylation arrays. R Packag version 060. 2016;

25. Xia C, Amador C, Huffman J, Trochet H, Campbell A, Porteous D, et al. Pedigree- and SNP-Associated Genetics and Recent Environment are the Major Contributors to Anthropometric and Cardiometabolic Trait Variation. PLoS Genet. 2016 Feb;12(2).

26. McCartney DL, Walker RM, Morris SW, McIntosh AM, Porteous DJ, Evans KL. Identification of polymorphic and off-target probe binding sites on the Illumina Infinium MethylationEPIC BeadChip. Genomics Data. 2016 Sep;9:22–4.

27. Hansen K. IlluminaHumanMethylation450kanno. ilmn12. hg19: annotation for Illumina’s 450k methylation arrays. 2016;

28. Min JL, Hemani G, Davey Smith G, Relton C, Suderman M. Meffil: efficient normalization and analysis of very large DNA methylation datasets. Bioinformatics. 2018 Dec;34(23):3983–9.

29. Du P, Kibbe WA, Lin SM. lumi: A pipeline for processing Illumina microarray. Bioinformatics. 2008 Jul;24(13):1547–8.

30. Chen YA, Lemire M, Choufani S, Butcher DT, Grafodatskaya D, Zanke BW, et al. Discovery of cross-reactive probes and polymorphic CpGs in the Illumina Infinium HumanMethylation450 microarray. Epigenetics. 2013;8(2):203–9.

31. Amador C, Huffman J, Trochet H, Campbell A, Porteous D, Wilson JF, et al. Recent genomic heritage in Scotland. BMC Genomics [Internet]. 2015;16(1):1–17. Available from: http://dx.doi.org/10.1186/s12864-015-1605-2

32. Houseman EA, Accomando WP, Koestler DC, Christensen BC, Marsit CJ, Nelson HH, et al. DNA methylation arrays as surrogate measures of cell mixture distribution. BMC Bioinformatics. 2012 May;13(1).

33. Joehanes R, Just AC, Marioni RE, Pilling LC, Reynolds LM, Mandaviya PR, et al. Epigenetic Signatures of Cigarette Smoking. Circ Cardiovasc Genet. 2016 Oct;9(5):436–47.

34. Min J, Hemani G, Hannon E, Dekkers K, Castillo-Fernandez J, Luijk R, et al. Genomic and phenomic insights from an atlas of genetic effects on DNA methylation. Nat Genet. 2020 Sep;25:81.

35. Wallace C. Statistical testing of shared genetic control for potentially related traits. Genet Epidemiol. 2013 Dec;37(8):802–13.

36. Guo H, Fortune MD, Burren OS, Schofield E, Todd JA, Wallace C. Integration of disease association and eQTL data using a Bayesian colocalisation approach highlights six candidate causal genes in immune-mediated diseases. Hum Mol Genet. 2015 Jun;24(12):3305–13.

37. Gay NR, Gloudemans M, Antonio ML, Abell NS, Balliu B, Park Y, et al. Impact of admixture and ancestry on eQTL analysis and GWAS colocalization in GTEx. Genome Biol 2020 211. 2020 Sep;21(1):1–20.

38. Hüls A, Czamara D. Methodological challenges in constructing DNA methylation risk scores. Epigenetics. 2020 Feb;15(1–2):1–11.

39. Barbu MC, Huider F, Campbell A, Amador C, Adams MJ, Lynall ME, et al. Methylome-wide association study of antidepressant use in Generation Scotland and the Netherlands Twin Register implicates the innate immune system. Mol Psychiatry 2021 [Internet]. 2021 Dec 8 [cited 2022 Feb 25];1–11. Available from: https://www.nature.com/articles/s41380-021-01412-7

40. Lyubenova A, Neupane D, Levis B, Wu Y, Sun Y, He C, et al. Depression prevalence based on the Edinburgh Postnatal Depression Scale compared to Structured Clinical Interview for DSM DIsorders classification: Systematic review and individual participant data meta-analysis. Int J Methods Psychiatr Res. 2021 Mar;30(1):30.

41. Barbu MC, Shen X, Walker RM, Howard DM, Evans KL, Whalley HC, et al. Epigenetic prediction of major depressive disorder. Mol Psychiatry. 2020 Jun;1–12.

42. Clark SL, Hattab MW, Chan RF, Shabalin AA, Han LKM, Zhao M, et al. A methylation study of long-term depression risk. Mol Psychiatry. 2019 Jun;25(6):1334–43.

**Figure 1.** Variance in MDD (indicated by R^2^ (%) on the y-axis) explained by each MS in (a) model 2 (covariates: age, sex, each MS’s corresponding phenotype) and (b) model 3 (covariates: model 2 + 4 lifestyle factors, BMI, smoking, pack years, and alcohol consumption) in GS (N=9,502) in logistic regression models (N=9,502). Where available, R^2^ is calculated for MS at different thresholds (educational attainment, smoking status, alcohol consumption). MW=methylome-wide (Bonferroni-corrected CpGs). * = p-value < 0.05; ** = p-value < 1x10^-5^.

**Figure 2.** Variance in MDD (indicated by R^2^ (%) on the y-axis) explained by each MS in (a) model 2 (covariates: age, 20 methylation PCs, and 5 cell types, each MS’s corresponding phenotype) and (b) model 3 (covariates: model 2 + 3 lifestyle factors, BMI, smoking, and alcohol consumption) in ALSPAC (N=565) in logistic regression models (N=565). Where available, R^2^ is calculated for MS at different thresholds (educational attainment, smoking status, alcohol consumption). MW=methylome-wide (Bonferroni-corrected CpGs). * = p-value < 0.05.

**Figure 3.** Receiver Operating Characteristic (ROC) curve indicating the sensitivity (*y*-axis) and specificity (*x*-axis) of environmental MS (Bonferroni-corrected CpGs) and MDD MS for MDD. The AUC estimates are indicated in black for each predictor in each graph, and the AUC estimate for MDD MS is indicated in red in all graphs.
